# Biological and functional relevance of CASP predictions

**DOI:** 10.1002/prot.25396

**Published:** 2017-10-17

**Authors:** Tianyun Liu, Shirbi Ish‐Shalom, Wen Torng, Aleix Lafita, Christian Bock, Matthew Mort, David N Cooper, Spencer Bliven, Guido Capitani, Sean D. Mooney, Russ B. Altman

**Affiliations:** ^1^ Department of Bioengineering Stanford University Stanford California; ^2^ Biomedical Informatics Training Program, Stanford University Stanford California; ^3^ Laboratory of Biomolecular Research Paul Scherrer Institute Villigen Switzerland; ^4^ Department of Biosystems Science and Engineering ETH Zurich 4058 Basel Switzerland; ^5^ Department of Biomedical Informatics and Medical Education University of Washington Seattle Washington; ^6^ Heidelberg University Heidelberg Germany; ^7^ Institute of Medical Genetics, Cardiff University United Kingdom; ^8^ National Center for Biotechnology Information, National Library of Medicine National Institutes of Health Bethesda Maryland; ^9^ Department of Biology ETH Zurich Zurich Switzerland

**Keywords:** protein structure prediction, functional relevance, microenvironment, binding site, CASP

## Abstract

Our goal is to answer the question: compared with experimental structures, how useful are predicted models for functional annotation? We assessed the functional utility of predicted models by comparing the performances of a suite of methods for functional characterization on the predictions and the experimental structures. We identified 28 sites in 25 protein targets to perform functional assessment. These 28 sites included nine sites with known ligand binding (holo‐sites), nine sites that are expected or suggested by experimental authors for small molecule binding (apo‐sites), and Ten sites containing important motifs, loops, or key residues with important disease‐associated mutations. We evaluated the utility of the predictions by comparing their microenvironments to the experimental structures. Overall structural quality correlates with functional utility. However, the best‐ranked predictions (global) may not have the best functional quality (local). Our assessment provides an ability to discriminate between predictions with high structural quality. When assessing ligand‐binding sites, most prediction methods have higher performance on apo‐sites than holo‐sites. Some servers show consistently high performance for certain types of functional sites. Finally, many functional sites are associated with protein‐protein interaction. We also analyzed biologically relevant features from the protein assemblies of two targets where the active site spanned the protein‐protein interface. For the assembly targets, we find that the features in the models are mainly determined by the choice of template.

## 1 INTRODUCTION

The ultimate goal of structure prediction is to provide insights into biological functions. However, it is difficult to quantify and benchmark the utility of protein structure prediction for functional inference.^1^ The biological function of a protein may have several different meanings; it can include catalyzing chemical reactions, transporting materials across the cell, receiving and sending chemical signals, or responding to stimuli and providing structural support. Most of these functions are realized by interacting with other proteins or small molecules. Therefore, interfaces between proteins, or interfaces between a protein and small molecules are critical to understanding function.

Official CASP structural assessments include global and local metrics that evaluate atomic level similarity of the structural features of proteins.^2^, ^3^, ^4^ The root mean square deviation (RMSD) was the first metric used in the CASP evaluations and it is still reported in the automatic evaluation system. The global distance test (GDT) score is effective for the automatic evaluation of predictions as it reflects absolute and relative accuracy of models for a wide range of target difficulty. In addition to GDT, several other similarity measures are used. Structural quality often tracks with functional quality, but the details of this correlation needs to be further explored.

The physicochemical environments within functional sites in experimentally solved structures are strongly associated with the functional properties of proteins. Therefore, a predicted structure that contains a similar physicochemical environment to an experimentally solved structure may be the most useful one for functional annotation. Previous studies have used a structural prediction protocol on a set of proteins and then compared the results of functional predictions with those from experimental structures.^5^, ^6^, ^7^, ^8^ In this work, we perform a systematic assessment that compares the ensembles of predictions of a target protein from different modeling algorithms to quantify the utility of predictions for inferring or recognizing function.

We address one simple question: to what extent do the CASP predictions accurately provide protein function information (compared to experimental structures)? To help define the term “protein function”, we asked the experimentalists why they were motivated to solve the structures. Based on the experimentalists' stated motivations, we defined regions or sites for assessment, including nine sites with known ligand binding (holo‐sites), nine sites that were expected or suggested by experimental authors to have small molecule binding (apo‐sites), and 10 sites containing motifs, loops, or key residues with important disease‐associated mutations. We evaluated the physical features of the predicted structure sites and the degree to which they shared similarity with the experimental structure sites. We previously developed PocketFEATURE (PF), an algorithm that evaluates similarity between two functional sites in terms of their physicochemical features.^9^, ^10^, ^11^, ^12^ As part of this work, we applied the PF algorithm to assess the extent to which physicochemical features that are observed in experimental structures can be replicated by predicted structures. We also analyzed features of quaternary structure assemblies in two oligomeric proteins and disease‐causing variants, which often play an important role in protein function.

## 2 RESULTS

### 2.1 Define sites

The biological rationale for determining a protein's structure provides a key perspective from which we evaluate the utility of predicted models. That is, what functional information should be provided by predictions from the viewpoint of the experimental authors? The answers we obtained from experimental authors varied in detail. Example include:

- “*First structure <in this family>…might help identify its function”;*
- *“Putative peptide‐binding site: D1154, F1147, I1162, M1163…”;*
- *“Interface: 46–48, 76–82, 104–120, 218–224”;*
- *“His204 of T0894 (CdiA‐CT) is involved in catalysis”;*
- *“Cys:His dyad as per other LD‐TP enzymes”;*
- *“It binds ADP”*.

Based on the answers, we defined three categories of functional sites by manually curating these answers and inspecting experimentally solved structures. The three categories are: (1) *nine holo sites*: pockets based on observed ligand binding in experimental structures, (2) *nine apo sites*: sites based on (a) critical residues provided by experimental authors, or (b) known motifs relevant to ligand or substrate binding, and/or (c) site finding algorithms, and (3) *ten critical patches*: patches centered at the key residues provided by experimental authors, including functionally critical residues, loops and mutations (Table 1 and Supporting Information Table S1). We evaluated the similarity of the three categories of pockets to the experimental sites.

**Table 1:** Twenty‐eight sites include nine known ligand binding site (holo, yellow), nine putative ligand binding site (apo, blue) and 10 critical patches surrounding key residues, motifs, or mutations (purple)^a^

Target ID	# points	Type	RMSD range (Å)	# servers	Classification
T0861	28	Holo/LLP	(0.945, 26.170)	42	TBM
T0863	8	Holo/CLR	(4.686, 284.38)	90	FM
T0873	24	Holo/FMN	(2.656, 109.89)	41	TBM
T0879	7	Holo/ZN/B	(4.213, 40.255)	39	TBM
T0889	21	Holo/SOR	(2.591, 44.685)	38	TBM
T0891	11	Holo/HEM	(2.314, 29.119	40	TBM
T0893	22	Holo/ADP	(9.251, 51.388)	43	TBM
T0910	27	Holo/ANP	(2.540, 45.013)	40	TBM
T0911	10	Holo/GCO	(4.377, 193.48)	105	TBM
T0880‐0	14	Apo	(8.492, 59.427)	108	FM
T0880‐1	13	Apo	(8.492, 59.427)	108	FM
T0894	19	Apo	(5.064, 68.908)	98	TBM
T0895	21	Apo	(4.353, 24.348)	110	TBM
T0896	23	Apo	(5.030, 150.96)	98	TBM
T0913	13	Apo	(3.955, 42.81)	108	TBM
T0917	73	Apo	(2.564, 44.06)	43	TBM
T0942	10	Apo	(2.992, 72.043)	92	TBM
T0947	25	Apo	(5.292, 33.782)	97	TBM
T0860	17	Motif	(2.654, 54.506)	42	TBM
T0864	11	Key residues	(10.34, 139.28)	101	FM
T0882	11	Key residues	(2.306, 25.381)	116	TBM
T0914	26	Key residues	(13.53, 127.45)	97	FM
T0915	14	Key residues	(5.42, 40.854)	108	FM
T0920‐0	31	Key residues	(3.213, 180.13)	39	TBM
T0920‐1	14	Key residues	(3.213, 180.13)	39	TBM
T0943‐1	9	Motif	(6.497, 77.188)	40	TBM
T0943‐2	10	Motif	(6.497, 77.188)	40	TBM
T0948	15–19	Mutation	(3.092, 36.111)	95	TBM

a The number of functional centers are in column 2. The types of sites are also noted in column 3, ligand IDs are marked when they are applicable. The RMSD ranges of all predicted structures are in column 4. The number of servers that made predictions on each target (site) is in column 5. Column 6 shows the target classification. Of the 28 sites, six sites are FM (free modeling) and 22 sites are TBM (template based modeling). The number of functional centers in each site is listed in column 2 (Specific resiude indexes are in Supporting Information Table S1). Note that we identified four critical patches surrounding four different mutations for T0948.

### 2.2 Overall assessments

**Figure 1:**
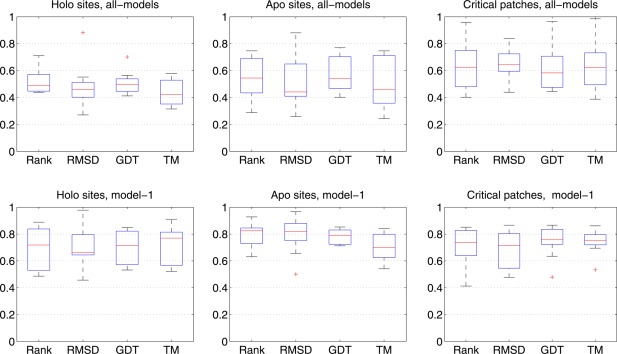
Correlation between functional assessments and CASP assessments are shown in box plots. All‐models are in top panel and model‐1 are at the bottom. The correlation coefficients for model‐1 are consistently higher than all‐models, suggesting that predictions with higher overall structure quality often have good functional features. The performance on holo sites is different from those on apo sites and critical patches (key residues): the overall (all‐models) correlation coefficients for holo sites are lower than that of apo sites or critical patches

We compared our assessment (using PF) on functional environment to the CASP assessments on overall structure quality (Figure 1). We aim to provide two references for users who are considering structural models for functional annotation: (1) Can model‐1 (the best in terms of their structure feature) provide robust functional insights? (2) Can the server (the average of all models) provide models with good functional features? PF measures the similarity between two sites in terms of their physicochemical features. The chosen official CASP assessments include the CASP ranking (see Methods), the global distance test (GDT), the template modeling score (TM), and root mean square deviation (RMSD). Figure 1 shows the correlation between PF and official CASP assessments (Analysis for individual target are available at https://simtk.org/projects/casp12funassess/.). In general, the correlation between PF and TM is lower than that between PF and GDT or PF and RMSD. This corresponds with the fact that TM is often considered as a more accurate measure of the quality of full‐length protein structures (compared with RMSD and GDT),^13^ while PF assess local characterization and may not reflect the quality of full structures.

CASP predictor teams could submit up to five models, ranked by their predicted quality. For each site, we either averaged scores over all submitted models (“all‐models”; Figure 1 top panel) or considered only the first model (“model‐1”; Figure 1 bottom panel). When we focus on the correlation between PF ranking and CASP ranking, the correlation coefficients for model‐1 are consistently higher than the all‐models average, indicating that predictions with higher overall structure quality often have good functional features (Supporting Information Table S2). For example, the correlation between PF‐ranking and CASP ranking for T0911 all‐models is about 0.4423 and that for model‐1 is 0.8878 (Predictor teams know which of their structures are likely the best.). It is interesting to note that the assessments on holo sites generally have lower correlation coefficients than those on apo sites and critical patches.

The correlation between our functional assessments and the structural assessments has two modes: (1) High correlation: predictions with high overall structure quality often have good local structure quality at their functional sites. This is reflected in the higher correlation on model‐1 assessments. (2) Low correlation: some predictions with excellent structure quality at local functional sites may not have good overall structure quality. For some targets, we found that PF‐scores do not track with the structural assessment, resulting in low correlation coefficients (Supporting Information Table S2).

Two servers (server‐220 GOAL^14^ and server‐005 Baker‐ROSETTASERVER^15^) made predictions on all 28 sites; this provides enough data to allow a comparison between servers (Supporting Information Table S3). Both servers showed fairly good performance in structural assessments. Table 2 shows our functional assessments and CASP assessments on model‐1 only. Using the CASP ranking, 12 of 28 model‐1 sites predicted by ROSETTASERVER were ranked in the top 30 models; whereas, 11 of 28 model‐1 sites predicted by GOAL were ranked in the top 30 models. Using functional assessments, 12 of 28 model‐1 sites predicted by ROSETTASERVER were ranked in the top 30 models; whereas, seven of 28 model‐1 sites predicted by GOAL were ranked in the top 30. Notably, for the site in T0920, PF ranked model‐1 from server 220 within the top 30 for its high similarity, even though its overall structure rank was 108.

**Table 2:** Comparison of GOAL (server 220) and Baker‐ROSETTASERVER (server 005)^a^

	Baker‐ROSETTASERVER		GOAL	
Target ID	PF‐zscore	CASP rank	PF‐zscore	CASP rank
T0861	–0.8359*	64	−0.3765	**29**
T0863	–2.5319*	**4**	−0.0974	**15**
T0873	−0.5657	**4**	−0.5067	32
T0879	−0.7310	28	−0.3296	49
T0889	–1.2986*	31	–1.1214*	36
T0891	−0.5022	82	−0.8670	**6**
T0893	−0.1618	**2**	−1.5486	73
T0910	−1.1330	**9**	−0.6228	31
T0911	−0.3728	130	−0.2285	60
T0880‐0	−0.5053	144	−1.2300	267
T0880‐1	0.3658	144	−0.1219	267
T0894	–2.2214*	**28**	−0.5339	204
T0895	−1.3542	136	−1.0160	**15**
T0896	−0.0480	**6**	−0.1733	98
T0913	−0.5680	**23**	−1.0342	100
T0917	–1.4857*	**5**	–1.4278*	**9**
T0942	0.8853	138	−0.3964	72
T0947	−0.7237	46	–1.4655*	**11**
T0860	–2.0525*	**1**	–1.9922*	**5**
T0864	−1.2324	26	−0.8253	300
T0882	–1.3716*	31	−1.2500	68
T0914	−1.0421	**12**	–1.9742*	230
T0915	–1.5203*	**17**	−0.8576	**29**
T0920‐0	–1.0713*	44	−0.5544	108
T0920‐1	−1.0446	44	–1.5482*	108
T0943‐1	0.0212	**2**	–2.1188*	**13**
T0943‐2	–1.7469*	**2**	−0.5068	**13**
T0948	–1.2990*	102	−0.5110	**25**

a These two servers made predictions on all 28 sites. We compared our functional assessments and CASP assessments on model‐1 generated by the two servers. Predictions that are selected as top 30 models by CASP structure assessment are highlighted in red (column 3 and column 5): 12 from Baker‐ROSETTASERVER and 11 from GOAL. Predictions that are selected as top 30 models (ranking of all‐models) by functional assessment are marked with * (column 2 and column 4): tweleve from Baker‐ROSETTASERVER and seven from GOAL. Note that the PF‐zscores for T0948 are the average of the four critical patches surrounding four different mutations (see Table 1).

### 2.3 Assessments on three types of sites

#### 2.3.1 Holo sites

The nine holo‐sites were defined based on the observed ligands in the experimental structures. The assessments compared the sites in predicted structures where the sites were not bound with ligands (apo) with the experimental structures where the sites were bound with ligands. The correlation coefficients between CASP rank and our functional assessments ranged from 0.44 to 0.71 (with four sites above 0.5) (Table 3). When comparing only model‐1, the correlation coefficients improved with averages of 0.49‐0.89 (with eight sites above 0.5). For all nine sites, the correlation coefficients between functional and structural assessments for model‐1 were higher than those for all‐models taken together. That is, for holo sites, the first ranked model (the best predicted model in terms of structure quality) contained better functional characterization.

**Table 3:** Correlation coefficient between functional assessment and CASP assessment (Rank)

Target	Rank All‐models	Rank Model‐1
T0861	0.7103	0.7182
T0863	0.5294	0.5518
T0873	0.4768	0.4861
T0879	0.5019	0.8785
T0889	0.4878	0.5269
T0891	0.4388	0.5297
T0893	0.4489	0.7801
T0910	0.6979	0.8240
T0911	0.4423	0.8878
*Average*	*0.5260*	0.6870
T0880‐0	0.5841	0.8242
T0880‐1	0.2886	0.8361
T0894	0.7466	0.7377
T0895	0.5451	0.6312
T0896	0.4436	0.8253
T0913	0.4835	0.8712
T0917	0.6812	0.7346
T0942	0.4067	0.9276
T0947	0.7191	0.7203
*Average*	*0.5442*	0.7898
T0860	0.4027	0.6878
T0864	0.6049	0.6229
T0882	0.7321	0.8510
T0914	0.9567	0.8503
T0915	0.4736	0.4125
T0920‐0	0.7562	0.7499
T0920‐1	0.4436	0.6219
T0943‐1	0.5040	0.7917
T0943‐2	0.8797	0.8388
T0948	0.6249	0.7380
*Average*	*0.6392*	0.7141

We evaluated six sites that had more than 10 predictions that were within 5 Å RMSD compared to the experimental structures (T0861, T0873, T0889, T0891, T0910, and T0911). For these six sites we selected the top 30 predictions based on functional assessments (Table 4 and Supporting Information section 2). We highlight one example to show how local functional environments can have characteristics that an overall structural assessment may not recognize. One example, T0891, is a heme binding protein. More than 70% of predictions have a GDT score better than 80. The experimental structure was solved with a heme‐binding molecule.

**Table 4:** Thirty predictions of T0891 selected by functional assessment (PF‐Zscores)^a^

Server ID and name	Model ID	PF‐Zscore	GDT	CASP rank
349 HHPred1	1	−2.0351	86.61	85
405 IntFOLD4	1	−1.7400	88.62	50
405 IntFOLD4	2	−1.7400	88.62	50
405 IntFOLD4	3	−1.7400	88.62	50
405 IntFOLD4	4	−1.7400	88.62	50
405 IntFOLD4	5	−1.7400	88.62	50
180 PhyreTopoAlpha	2	−1.7263	82.59	118
236 MULTICOM‐CONSTRUCT	3	−1.6528	89.73	28
425 FALCON_TOPOX	3	−1.6366	89.29	37
313 HHGG	1	−1.6080	87.28	74
220 GOAL	2	−1.4660	91.74	1
287 MULTICOM‐CLUSTER	1	−1.3041	90.18	17
446 YASARA	5	−1.2917	91.07	4
345 MULTICOM‐NOVEL	4	−1.2332	89.51	31
275 slbio	4	−1.2120	87.72	66
313 HHGG	5	−1.2095	86.38	89
236 MULTICOM‐CONSTRUCT	1	−1.1696	90.18	17
236 MULTICOM‐CONSTRUCT	5	−1.1472	87.5	72
425 FALCON_TOPOX	5	−1.1472	89.06	41
446 YASARA	1	−1.0962	88.62	50
236 MULTICOM‐CONSTRUCT	4	−1.0937	90.62	9
382 RBO_Aleph	5	−1.0750	86.16	95
026 chuo‐u2	5	−1.0501	79.02	129
380 chuo‐u‐server	5	−1.0501	79.02	129
258 MUfold1	4	−1.0389	89.51	31
077 FALCON_TOPO	2	−1.0376	88.17	62
382 RBO_Aleph	1	−1.0065	86.61	85
250 Seok‐server	2	−0.9667	90.18	17
077 FALCON_TOPO	3	−0.9542	88.39	58
166 FFAS03	1	−0.9156	81.03	122

a Model ID was from lables by the predictors. CASP official assessments are in column 4 and column 5.

**Figure 2:**
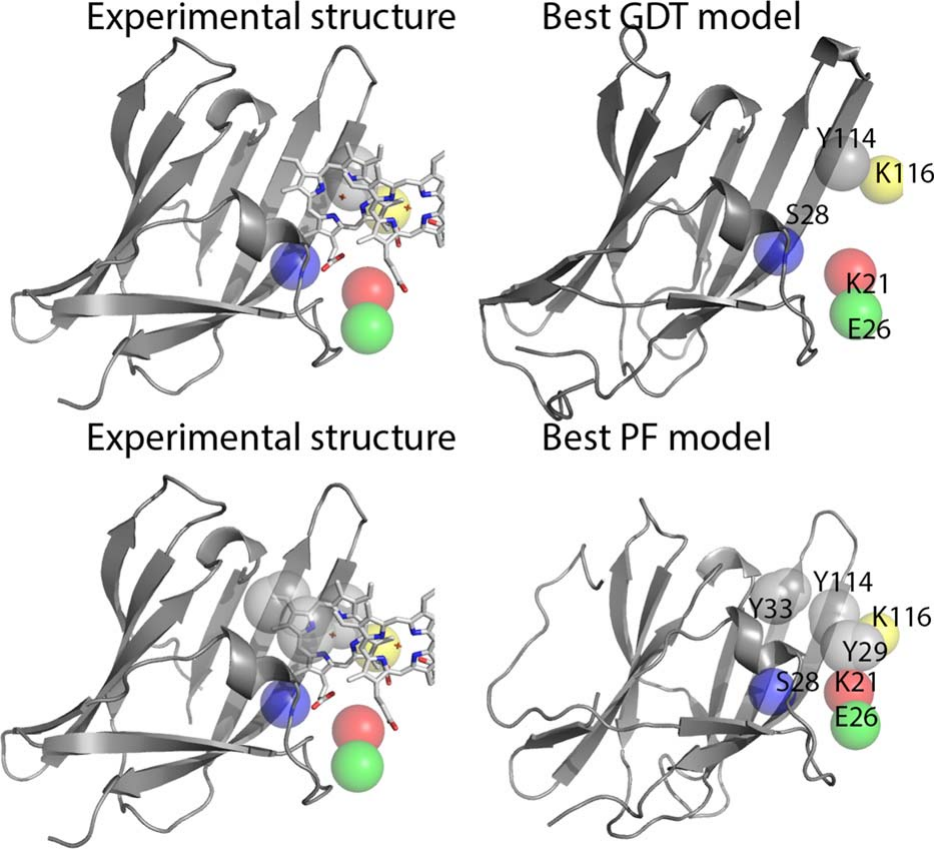
The experimental structure of T0891 has a heme binding site. Local features in the best PF ranked model with those observed in the best structure model (best GDT model). The model‐2 from server GOAL (220–2) has the best GDT score (91.74) among all the predictions, while its PF‐zscore is −1.466 (A more negative PF‐zscore suggests better similarity.) The model‐1 from HHPred (349–1) was ranked best by our functional assessment with a PF‐zscore of −2.035, but its GDT score is 86.61. When aligning microenvironments surrounding the heme‐binding site, the best structural model (220–2) shares five similar microenvironments with the experimental structure. The best PF ranked model shares seven similar microenvironments with the experimental structure

For T0891, we compared the local features in the best PF ranked model with those observed in the best structure model (best GDT model) in Figure 2. The model‐2 from server GOAL (220–2) has the best GDT score (91.74) among all the predictions, while its PF‐zscore is −1.466. PF estimates similarities by matching similar microenvironments between two sites. Microenvironment refers to the local, spherical region in the protein structure that may encompass residues discontinuous in sequence and structure (See method). A higher number of matched microenvironments and a more negative PF‐zscore suggest better similarity. The model‐1 from HHPred (349–1) was ranked best by our functional assessment with a PF‐zscore of −2.035, but its GDT score was 86.61. When aligning microenvironments surrounding the heme‐binding site, the best structural model (220–2) shared five similar microenvironments with the experimental structure. We noticed that the secondary structures near the binding site were slightly different from those in the experimental structure. The top PF ranked model matched an additional two microenvironments to the experimental structure due to better positioning of the heme‐binding motifs.

#### 2.3.2 Apo sites

The nine apo‐sites were defined based on the information provided by experimental authors combined with a ligand‐binding site searching program (Fpocket^16^). The assessments compared sites in predicted structures (apo) to the corresponding sites in experimental structures (apo). The correlation coefficients between CASP rank and our functional assessments ranged from 0.28 to 0.75 (with five sites above 0.5) (Table 3). When comparing only model‐1, the correlation coefficients improved with averages of 0.63‐0.87 (with all nine sites above 0.5). For eight of the nine sites, the correlation coefficients between functional and structural assessments for model‐1 were higher than those for all‐models. Notably, the average correlation between functional assessments and CASP assessments was higher than that for holo sites (Figure 1 and Table 3).

**Figure 3:**
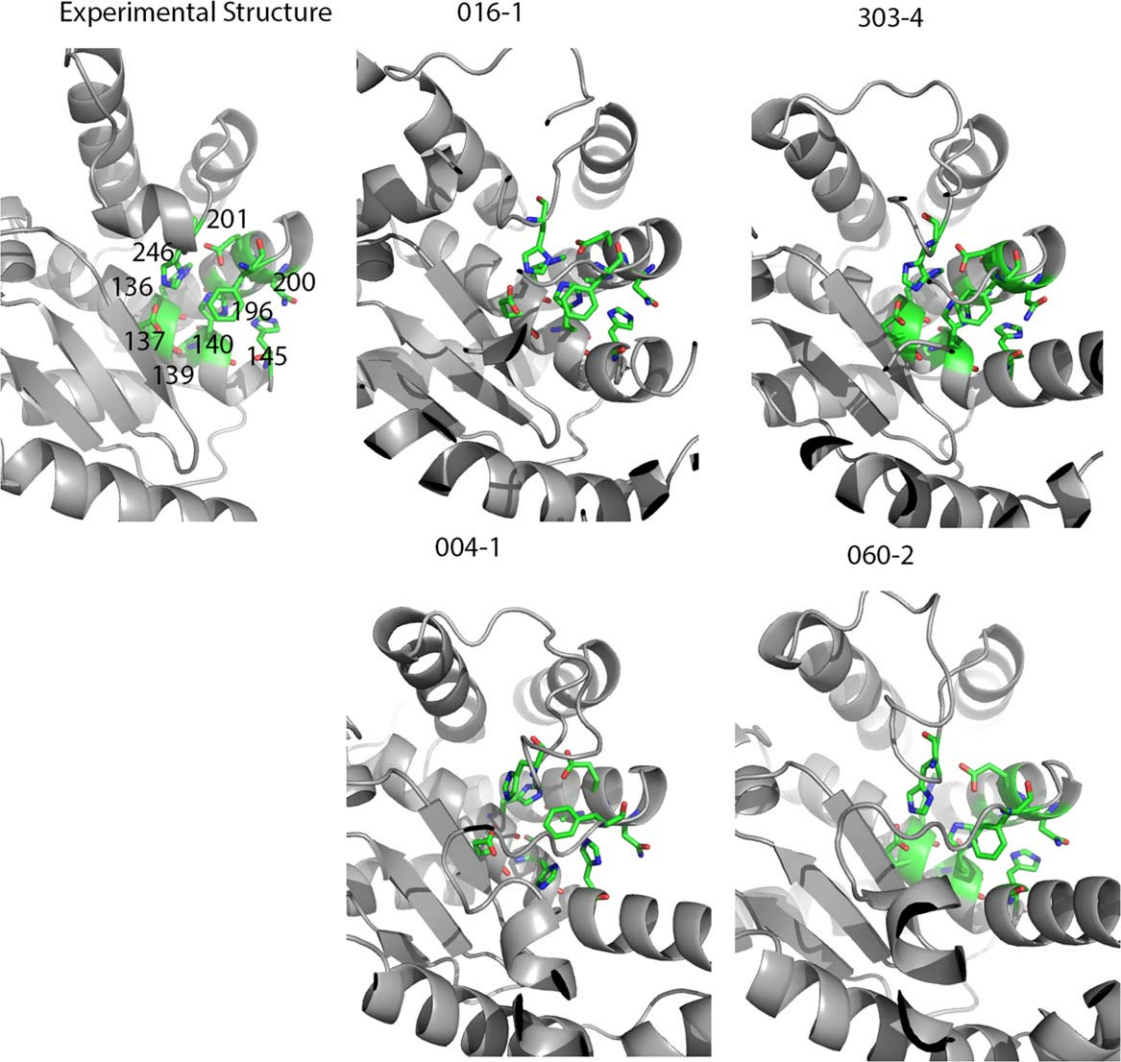
T0942 has an HEXXH motif of metalloproteinase, identified by sequence analysis. The motif forms a histine enriched pocket that may bind Zinc (residue index 145 H, 140 H, 136 H, 246 H). Other residues near this motif include 137E, 139 S, 196 F, 200 N, 201E. We compare the side‐chains near these four residues between experimental structures, the best GDT ranked model‐1 (004–1, GDT 46.3, PF‐zscore −0.93), the best GDT ranked all‐models (060–2, GDT 54.5, PF‐zscore −1.01), the best PF ranked model‐1 (016–1, GDT 38.6, PF‐zscore −1.50) and the best PF ranked all‐models (303–4, GDT 39.8, PF‐zscore −1.79). The best PF‐ranked models share similar side‐chain arrangements with the experimental structures, while the best GDT‐ranked models do not

**Figure 4:**
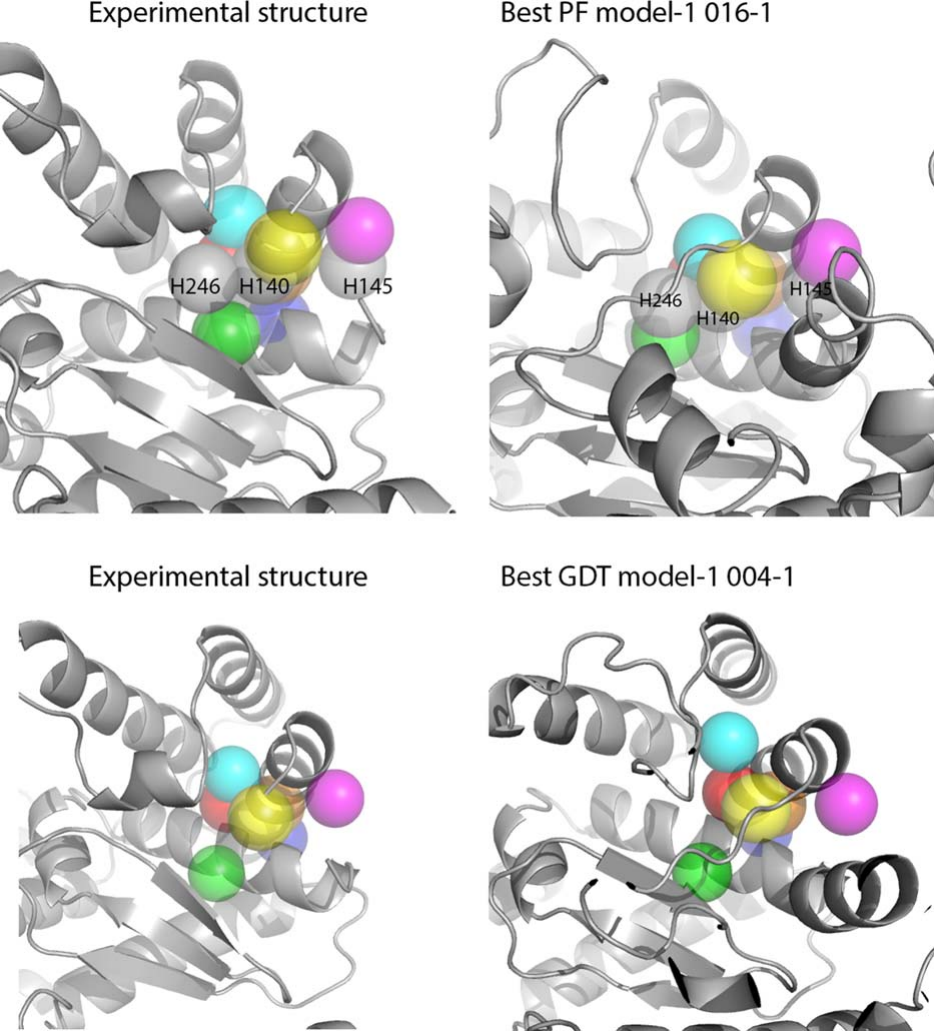
We compare the microenvironment alignments between experimental structures, the best GDT ranked model‐1 (004–1, GDT 46.3, PF‐zscore −0.93), and the best PF ranked model‐1 (016–1, GDT 38.6, PF‐zscore −1.50). The best GDT model‐1 has seven aligned microenvironments. The best PF‐scored model 016–1 has 10 microenvironments aligned. The three additional aligned microenvironments observed in the best PF model‐1 are colored in grey: H246, H140, and H145, which are the key element of metaloproteinase motifs

We evaluated six sites that had >10 predictions were within 5 Å RMSD compared to the experimental structures (T0942, T0894, T0895, T0896, T0913, and T0917). For the six sites, we selected the top 30 predictions based on functional assessments (See Supporting Information section 2). Using T0942 as an example, we demonstrated how functional assessments capture local physicochemical properties (Figure 3). T0942 has an HEXXH motif of metalloproteinase, identified by sequence analysis. The motif forms a histidine‐enriched site (residue 145 H, 140 H, 136 H, 246 H) that may bind zinc. Other residues near this motif include 137E, 139 S, 196 F, 200 N, 201E. We compared these side‐chains from experimental structures, including the best GDT ranked model‐1 (004–1, GDT 46.3, PF‐zscore −0.93), the best GDT ranked all‐models (060–2, GDT 54.5, PF‐zscore −1.01), the best PF ranked model‐1 (016–1, GDT 38.6, PF‐zscore −1.50), and the best PF ranked all‐models (303–4, GDT 39.8, PF‐zscore −1.79). The top PF‐ranked models share similar side‐chain arrangements with the experimental structures whereas, the best GDT‐ranked models did not. We compared the microenvironment alignments between the experimental structures, the best GDT ranked model‐1 (004–1, GDT 46.3, PF‐zscore −0.93), and the best PF ranked model‐1 (016–1, GDT 38.6, PF‐zscore −1.50). The best GDT model‐1 had seven aligned microenvironments. The best PF model‐1 had 10 aligned microenvironments. The three additional aligned microenvironments observed in the best PF model‐1 are colored in grey: H246, H140, and H145, which are the key elements of metalloproteinase motifs (Figure 4 and Table 5).

**Table 5:** Thirty predictions of T0942 selected by functional assessment^a^

Server ID and name	Model ID	PF—Zscore	GDT	CASP rank
303 wfMESHI‐TIGRESS	4	−1.7898	39.79	85
382 RBO_Aleph	5	−1.7473	34.17	243
405 IntFOLD4	3	−1.7212	41.73	54
320 raghavagps	3	−1.7212	41.73	54
411 Pcomb‐domain	4	−1.6958	39.79	85
042 Elofsson	3	−1.6280	38.11	151
016 FFAS‐3D	1	−1.4968	38.57	133
073 Wallner	1	−1.4431	43.28	41
236 MULTICOM‐CONSTRUCT	1	−1.4416	38.05	155
067 wfRstta‐PQ2‐Seder	1	−1.4386	38.7	127
028 M4T‐SmotifTF	1	−1.4386	38.7	127
079 iFold_1	1	−1.4386	38.7	127
382 RBO_Aleph	1	−1.4140	34.62	238
464 tsspred2	1	−1.3879	39.99	79
486 TASSER	5	−1.3879	39.99	79
464 tsspred2	4	−1.3857	39.86	83
239 wfAll‐Cheng	5	−1.3782	39.41	103
303 wfMESHI‐TIGRESS	2	−1.3782	39.41	103
464 tsspred2	3	−1.3395	37.92	165
243 Seok‐refine	4	−1.3245	39.53	99
411 Pcomb‐domain	5	−1.3163	39.79	85
405 IntFOLD4	4	−1.2977	38.18	144
382 RBO_Aleph	3	−1.2940	34.88	235
396 PML	1	−1.2820	39.73	91
079 iFold_1	3	−1.2791	38.11	151
166 FFAS03	1	−1.2791	38.11	151
243 Seok‐refine	2	−1.2761	39.15	109
102 Kiharalab	4	−1.2560	39.6	97
479 Zhang‐Server	1	−1.2560	39.6	97
067 wfRstta‐PQ2‐Seder	2	−1.2351	39.73	91

a (PF‐Zscores). Model ID was from lables by the predictors. CASP official assessments are in column 4 and column 5.

#### 2.3.3 Critical patches

The 10 critical patches were defined based on the information provided by experimental authors and resources, such as sequence analysis and a literature review. We compared the microenvironments surrounding the patches in predicted structures with those in experimental structures. Table 3 shows the correlation coefficients between CASP rank and our functional assessments ranging from 0.40 to 0.96 (with seven sites above 0.5). When comparing only model‐1, the correlation coefficients ranged from 0.41 to 0.85 (with eight sites above 0.5). In this category, model‐1 (the best predicted model in terms of structure quality) and other models have similar levels of functional characterizations.

We evaluated four sites that had >10 predictions that were within 5 Å RMSD compared to the experimental structures (T0860, T0882, T0920‐0, T0920‐1). For these four sites we selected the top 30 predictions based on functional assessments (Supporting Information section 2). In this category, functional information is often not available to predictors (in contrast to ligand binding sites); hence, we observe greater deviation between structural quality and functional quality. For example, when we ranked model‐1 for the critical patch T0920‐1, the best functionally characterized prediction was 220–1 (GOAL), whose official CASP rank was 108 in terms of its overall structural quality (Table 2 and Supporting Information section 2).

**Figure 5:**
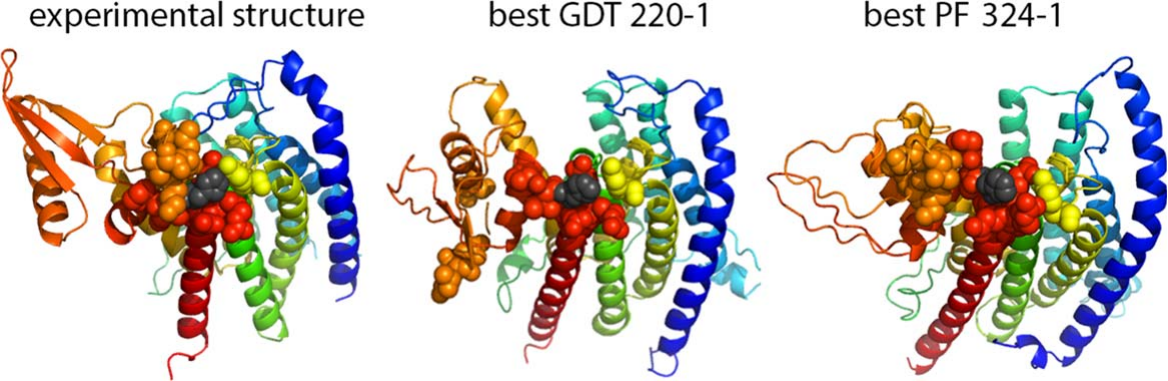
Analysis on the critical patch on T0945 (center at SNP 376 H, colored in black). The patch include residues: 245, 308, 312, 313, 314, 315, 374, 375, 376, 377, 378, 379, 380, 381, and 83, which form a tight cluster near 376 H (left). The 15 residues surrounding this SNP are the microenvironments associated with the functional effects of the SNP. However, in the best GDT model (best GDT 220–1, GDT 59.27, PF‐zscore −1.32) these microenvironments are not clustered near 376 H. This is because one of the key loops was predicted away from the functional center. In the best PF ranked model (best PF model, 324–1, GDT 54.07, PF‐zscore −1.55), the corresponding microenvironments form one cluster, with the functional loops predicted in the right position

We applied PocketFEATURE to analyze patches surrounding mutations in two targets: T0948 (four patches) and T0945 (20 patches). The four patches in T0948 cluster together and were treated as one functional site for overall assessment on critical patches, as discussed above (Tables 1, 2, 3). We analyzed the 20 mutation patches and found that the functional ranking tracks with the overall structure quality, but with great deviations (Supporting Information Table S11). Figure 5 shows one patch surrounding a single nucleotide polymorphism (SNP) 376 H. The patch included residues: 245, 308, 312, 313, 314, 315, 374, 375, 376, 377, 378, 379, 380, 381, and 83, all of which form a tight cluster near 376 H (left, experimental structure). The 15 residues surrounding this SNP are the microenvironments associated with the functional effects of mutations. However, in the best GDT model (best GDT 220–1, GDT 59.27, PF‐zscore −1.32) these microenvironments are not clustered near 376 H because one of the key loops was not predicted near the functional center. In the top PF ranked model (best PF model, 324–1, GDT 54.07, PF‐zscore −1.55), the corresponding microenvironments form one cluster with the functional loops predicted in the correct position, even though the secondary structures in the neighboring domain are not correctly predicted.

### 2.4 Assessments from other research groups

#### 2.4.1 Functional prediction in dimeric targets (Capitani research group)

Two target assemblies contained a pocket at the protein interface: CckA histidine kinase (T0893), and STRA6 receptor (T0930). CckA is a histidine kinase, a dimeric bifunctional enzyme mediating both phosphorylation and dephosphorylation of downstream targets.^17^ The most important features of the quaternary structure are (1) the conserved, exposed histidine residue, which acts as a phosphate acceptor during autophosphorylation, (2) the connectivity of the four helices of the dimerization and histidine phosphotransfer (DHp) domain and (3) the relative position of the catalytically active (CA) domain to the DHp domain.^18^ A total of eight groups submitted dimeric models with acceptable oligomeric quality for T0893 (Supporting Information Figure S2 and Table S9). These were manually inspected for the presence of the three features. All the models exposed the phosphate acceptor histidine, four models correctly reproduced the connectivity of the four helices of the DHp domain, and two models predicted the correct position of the CA domain for *cis* autophosphorylation. However, no model included all three features.

STRA6 is a dimeric integral membrane receptor for retinol uptake that associates with the retinol binding protein (RBP) and translocates the retinol molecule into the lipid bilayer.^19^ The two features of the STRA6 receptor dimer important for its function are the geometry of the cleft in the dimeric interface, which bends the outer membrane outwards, and the coordination of residues from both subunits to create the RBP‐binding motif.

Unfortunately, STRA6 had no sequence similarity to any known membrane transporter, channel, or receptor at the time of the CASP12 experiment, and the prediction of its tertiary structure and assembly was unsuccessful. Therefore, no predictions were of sufficient quality to provide biologically relevant information about the function.

#### 2.4.2 Predictions at missense mutation sites (Mooney research group)

To evaluate whether structure predictions can be interpreted as an indicator of the pathogenicity status of missense mutations, we assessed secondary structure and solvent accessibility predictions. The mutation databases ClinVar^20^ and HGMD^21^ were utilized to obtain a total of 20 unique, non‐synonymous pathogenic variants and 64 variants of unknown clinical significance (VUS) for the target region T0945 of the DPAGT1 protein. Being an essential part of N‐glycosylation, the observed DPAGT1 mutations are linked to myasthenia and myopathy [Selcen 2014] and limb‐girdle congenital myasthenic syndrome with tubular aggregates.^22^ In addition, DPAGT1 is involved in disturbing intercellular adhesion in oral cancer.^23^ To measure prediction accuracy at pathogenic variants and VUS, we analyzed the following five metrics for variant‐affected residues: (1) The standard deviation of predicted relative (RelAcc) and absolute solvent accessibility. (2) The RMSD between predicted and correct relative and absolute solvent accessibility (AccErr). (3) The fraction of correctly and incorrectly predicted secondary structure as reported by DSSP [Kabsch and Sander 1983]. (4) The fraction of correctly and incorrectly predicted exposure statuses. Based on its relative solvent accessibility a residue is considered buried (RelAcc < 0.09), intermediate (0.09 ≥ RelAcc < 0.36), or exposed (RelAcc ≥ 0.36).^24^ (5) The distribution of pathogenic variants in highly conserved residues as reported through ConSurf.^25^

For residues affected by pathogenic variants the average RMSD and standard deviation of relative solvent accessibility is 0.14 and 0.20, respectively (Supporting Information Figure S3). We did not find a significant difference between these values and the according metrics for residues affected by VUS. Hence, a suspected correlation between prediction accuracy of an ensemble of structure predictors and a variant's pathogenicity could not be established.

Comparing the 20 identified pathogenic variants in T0945 with all 488 VUS, the absolute solvent accessibility values for both groups distribute similarly (Median: 0.16 and 0.19, STD: 37.76 and 34.20). After categorizing residues in buried, intermediate, or exposed, the exposure status of 34.0% of all buried, 30.4% of all exposed, and 58.0% of all intermediate variants are incorrectly predicted. Wrongly predicted exposure states for pathogenic variants/VUS in DPAGT1 are distributed as follows: 31.17/26.40% (buried), 45.48/64.71% (intermediate), and 33.45/42.64% (exposed). In general, the AccError does not correlate with GDT. This suggests that the prediction quality of a single missense mutation is not reflected in the overall quality of the structures.

## 3 DISCUSSION

### 3.1 Physicochemical properties in microenvironments carry functional critical information

We have previously reported a system, FEATURE,^12^ for representing protein “microenvironments”, as statistical descriptions of physicochemical and structural features in a sphere volume of 7.5 Å radius. A single ligand site is often comprised of between 10 to 20 microenvironments, each centering on one of the key residues. PocketFEATURE employs a matching system that aligns similar microenvironments, or physicochemical properties, between sites or even entire proteins (instead of sequence alignments). PocketFEATURE can distinguish statically and dynamically between similar sites, between homologs,^26^ and even between unrelated proteins.^9^ PocketFEATURE is able to distinguish aspects of the drug‐binding pocket in FtsZ structures from different species that are not evident with other comparison methods such as RMSD. PocketFEATURE can also detect the effects of mutations in protein pockets.^26^ In addition, it can detect key functional changes driving molecular dynamic trajectories. Our analysis is based on the evidence that PocketFEATURE can distinguish more finely grained physicochemical differences associated with protein function—including ligand binding or mutation effects between sites with very similar structure properties. Of course other methods (SiteCompare^27^ and SMAP^28^) that share similar characteristics could also be used.

### 3.2 Differential performance on holo and apo sites

We observed that predictions using holo sites differ in quality from those using apo sites and critical patches. In all‐models assessments, the correlation coefficients for holo sites are lower than the other two categories. Given a sequence with templates that have bound ligand(s), predictors generate “apo models” that do not take the ligand information into account (They may consider ligand information implicitly if they use templates that contain a bound ligand). Experimental structures solved with a bound ligand often have different physicochemical characterizations (to form non‐covalent contacts for ligand binding). Therefore, when comparing apo predictions with experimental holo structures, we expect lower similarities at these sites. Our results confirm that the quality of local sites (as measured by the similarity to experimental sites) may not be reflected in the overall structure quality. However, local similarity to the experimental sites is useful in deriving biological and functional information from predicted models. Therefore, we looked for methods and servers that could predict holo sites well (Supporting Information section 2 Tables S4 and S7). Predictions from servers Multicom‐construct^29^ and IntFOLD4^30^ performed well for all six holo sites. These methods likely employed algorithms that benefited from ligand‐binding template information and for modeling.

### 3.3 Predicting function in protein assemblies

This iteration of CASP received an encouraging number of oligomeric predictions. Correctly predicting the stoichiometry and protein‐protein interfaces for a protein complex can be extremely important for understanding the biological function of a protein. The two cases presented here represent the particularly difficult task of predicting functions that arise through the interaction of multiple subunits. In this context, the failure of any groups to adequately model the STRA6 translocation path is unsurprising.

The lack of CckA models containing both the correct 4‐helical bundle topology and the *cis* binding affinity can be explained by considering the available templates. The structure of CckA has a unique DHp and CA domain arrangement that was not shared by any of the available templates. Thus, models were biased by the choice of template toward incorrect domain orientations.

### 3.4 Method limitations

In general, functional utility correlates with the quality of structure predictions, but there are interesting deviations. Predictions with higher overall structural quality (model‐1) often have good functional utility. However, some predictions with good structural quality may not have the best local functional sites, and sometimes these are significantly worse. Using PocketFEATURE to evaluate physicochemical properties at local functional sites provides reasonably good discrimination between predictions with similar structural quality.

The major uncertainty of our assessment originates from the ill‐defined nature of functional sites and functional centers. Even with communication with the experimentalists, it was difficult for us to achieve an undisputed functional site definition. In future CASPs, it would be useful to have a more structured and systematic procedure to retrieve biological relevance from the experimental contributors. Nonetheless, our evaluations still suggest substantial biological utility despite some partial site definitions. We found that scoring a SNP alone (very local) does not track with the overall structure quality, but scoring patches surrounding a SNP provide more insights into functional relevance (Figure 5). We also compared defined site residues' PF assessments with local RMSD measurements (Supporting Information Figure S1). The local RMSD correlate well with overall RMSD, but not PF‐scores, suggesting that PF evaluates physicochemical properties beyond structure features. In addition, the local estimation also depends on the site definition, which is one of the key limitations of functional assessment methods.

## 4 METHODS

### 4.1 Define and describe sites for evaluation

We emailed to ask experimental authors: (1) Why did you decide to solve the structure of this target? (2) Can you briefly describe the function of the target? (3) Are there any of these types of sites: enzymatic active sites, small molecule binding pockets, protein interaction sites, nucleic acid interaction sites, mutated sites, critical loops, domain boundaries, other critical areas.

Based on their answers (on 43 targets), we selected 25 targets for which sufficient information was provided. We assigned targets to three groups to assess their utility in functional annotation. (1) Holo sites: defined sites based on observed ligand binding in experimental structures. (2) Apo sites: defined sites based on critical residues provided by experimental authors or known motifs relevant to active sites. We employed a patch‐searching algorithm F‐Pocket for initial screening and then manually selected sites based on experimental authors' answers. (3) Critical patches: defined patches centered at the critical residues (including SNPs) provided by experimental authors.

Our previous work demonstrates that functional properties of a critical region can be extracted by describing their physicochemical environments.^12^ We have developed the FEATURE system that computes a set of 80 physicochemical properties collected over six concentric spherical shells (total 480 properties = 80 properties × 6 shells) centered on a predefined functional center.

### 4.2 Compare sites in predictions and experimental structures

PocketFEATURE contains two essential modules to evaluate and compare physicochemical properties of a single or a cluster of functional centers.^9^ The two modules are:

1. Given two centers (can be an atom, or average coordinates of multiple atoms) from two structures, we use the term “microenvironment”to refer to the local, spherical region in the protein structure that may encompass residues discontinuous in sequence and structure. We then measure the similarity between the two microenvironments by a Tanimoto‐based approach (see Supporting Information: method description).
2. Given two binding sites (or two clusters of functional centers), we exhaustively calculate the similarities between all permissible microenvironment‐pairs. We then search for the mutual most similar microenvironment‐pairs between two binding sites and assign alignments and similarity scores between the two binding sites (see Supporting Information Section 4: method description).

We applied the two modules of PocketFEATURE to assess the physicochemical environments of a single or cluster of functional residue centers.

For apo and holo sites, the challenge was to evaluate how well the binding sites are predicted, in terms of the pocket's physicochemical environments, given the quality similarities of the overall predicted structures. We applied PocketFEATURE to compare experimental sites to the corresponding microenvironment centers in the predicted structures. The similarity between the two sites provides an estimate of the probability of a ligand binding to the predicted site, which is the biological relevance of apo and holo sites.

For *critical patches*, the challenge is to evaluate how well the critical regions associated with the functions of interest are predicted (compared with the experimental structures), in terms of the overall physicochemical environments of the critical regions. We adopted the procedure above with modifications based on the shape and the size of the critical regions.

### 4.3 Compare functional assessments and CASP structure assessments

CASP predictions were downloaded from assessors' section of the CASP website. In the assessors' section, under the predictions folder, there was a gziped folder for each target containing all predictions from all servers. CASP rankings and other measurements, including GDT, TM, and RMSD (official assessments), were obtained from the CASP website (CASP12 result section).

We performed two assessments: “all‐models” and “model‐1”. For each target, each prediction server may generate one to five models, with their best model labeled as model‐1 before submitting to CASP assessment committee. For “all‐models”, we calculated PocketFEATURE zscores (PF‐zscore) of all server models for each of the 28 sites. Specifically, scores of all predictions of a given target from each server were treated as independent predictions. PocketFEATURE scores across these models for each site were then normalized to obtain the zscores using the scipy.stats.zscore package. For “model‐1”, we apply the same procedure to models labeled with model‐1 by the predictors.

## AUTHOR CONTRIBUTIONS

T.L. and R.B.A. designed and led the assessments. T.L., S.I., and W.T. performed the functional site assessments. A.L. performed the protein assembly assessments. C.B. performed the missense assessment. G.C. advised on assembly assessments. M.M., D.N.C., and S.D.M. advised on missense mutations. T.L. led the manuscript writing. T.L., R.B.L., S.I., W.T., A.L., C.B., and S.B. contributed writing and editing.

## Supporting information

Additional Supporting Information may be found online in the supporting information tab for this article.

Supporting Information
