# Supplementary material for: Biological and functional relevance of CASP predictions
Source: Proteins. 2017 Oct 17;86(Suppl Suppl 1):374–86. doi: 10.1002/prot.25396 (PMC5820171; doi:10.1002/prot.25396)
Supplement: Supplementary file 1 — Supporting Information [file PROT-86-374-s001.docx]

**Section 1: Overall assessment**

Table S1A. 28 sites for assessment. (1) Yellow: Ligand co-crystalized in experimental structures. Holo structure; (2) Blue:Known small molecule binding sites or catalytic sites. Apo structures. (3). Purple: motifs, loops and key residues involved in function. (4) Red: 4 mutations in one site of T0948.

| Target ID | Strategy | Residues | #  pts | Ligand | #  pred. | # ser | RMSD range |
| --- | --- | --- | --- | --- | --- | --- | --- |
| T0861 | ligand in the experimental structure | 38, 40, 41, 42, 43, 45, 46, 71, 72, 73, 76, 143, 149, 153, 175, 176, 177, 178, 179, 180, 181, 182, 229, 230, 273, 300, 301, 306 | 28 | LLP/A/42 | 181 | 42 | (0.945, 26.170) |
| T0863 | ligand in the experimental structure | 416, 419, 420, 423, 424, 516, 519, 520 | 8 | CLR/F/1 | 391 | 90 | (4.686, 284.38) |
| T0873 | ligand in the experimental structure | 165, 166, 180, 182, 183, 184, 185, 197, 199, 202, 203, 204, 234, 235, 237, 244, 324, 326, 333, 336, 337, 341, 402 | 24 | FMN/B/1 | 177 | 41 | (2.656, 109.89) |
| T0879 | ligand in the experimental structure | 24, 25, 73, 77, 79, 114, 183 | 7 | ZN/B/1 | 166 | 39 | (4.213, 40.255) |
| T0889 | ligand in the experimental structure | 92, 140, 141, 142, 147, 149, 150, 153, 183, 184, 185, 186, 188, 190,194, 240, 242 | 21 | SOR/A/301 | 162 | 38 | (2.591, 44.685) |
| T0891 | ligand in the experimental structure | 21, 24, 26, 28, 29, 33, 114, 116 | 11 | HEM/B/155 | 172 | 40 | (2.314, 29.119) |
| T0893 | ligand in the experimental structure | 131, 132, 135, 176, 178, 179, 180, 181, 189, 194, 195, 196, 197, 202, 203, 204, 205, 206, 207, 208, 231, 233 | 22 | ADP/A/600 | 186 | 43 | (9.251, 51.388) |
| T0910 | ligand in the experimental structure | 40, 41, 42, 43, 44, 45, 46 ,48 ,62, 64, 83, 96, 112, 114, 115, 116, 119, 158, 160, 163, 165,175, 176, 178, 179, 195, 323 | 27 | ANP/A/700 | 165 | 40 | (2.540, 45.013) |
| T0911 | ligand in the experimental structure | 68, 123, 126, 358, 371, 374, 375, 377, 378 | 10 | GCO/C/1 | 463 | 105 | (4.377, 193.48) |
| T0880-0 | Manually define 2 pockets based on (1) Six residues from experimental clues: Asn 647, Tyr650, Glu652 and residues 679-682,  (2) fpocket result | 647, 650, 652, 679-682 | 14 |  | 484 | 108 | (8.492, 59.427) |
| T0880-1 | Manually define 2 pockets based on (1) Six residues from experimental clues: Asn 647, Tyr650, Glu652 and residues 679-682  (2) fpocket result | 53, 56, 58, 83, 85, 86, 87, 88, 89, 90, 91, 173 | 13 |  | 484 | 108 | (8.492, 59.427) |
| T0894 | The key residues are HIS 204, ASP 48, SER 201, pocket_1 from fpocket match well with the key residues. (radius = 8 Angstrom, threshold=15 residues) | T0894: 188, 189, 193, 199, 201, 202, 203, 204, 205, 261, 262, 263, 308, 310, 320, 321,322, 323 | 19 |  | 440 | 98 | (5.064,  68.908) |
| T0895 | fpocket pocket_1. Radius based method was used to slightly extend the pocket (radius = 8 Angstrom, threshold=15 residues) | T0895: 46, 47, 48, 49, 50, 51, 52, 53, 63, 70, 71, 72, 73, 74, 76, 77, 78, 81, 82, 83 | 21 |  | 505 | 110 | (4.353, 24.348) |
| T0896 | Enzyme around CYS dyad. "Structure of LdtMt2, an L, D-transpeptidase from Mycobacterium tuberculosis." Acta Crystallographica: Biological Crystallography 2013. Pocket center at key residues: HIS 155, CYS 147, CYS 153 | T0896: 131, 144, 145,146,147, 148, 149, 150, 151, 152, 153, 154, 155, 156, 339, 437, 438, 439, 440, 471, 472, 473 | 23 |  | 437 | 98 | (5.030, 150.964) |
| T0913 | fpocket_5 was used directly to define residues, the full pocket was then extracted based on the residue list | T0913: 209, 210, 273, 274, 320, 321, 363, 368, 371, 64, 65, 66, 67 | 13 |  | 484 | 108 | (3.955, 42.805) |
| T0917 | fpocket_0 was used directly to define residues, the full pocket was then extracted based on the residue list | 104-109, 111, 136, 157, 158, 160, 161, 163, 166, 168, 170, 172, 179, 181, 198, 201, 202, 203, 206, 209, 210, 213, 217, 273, 276, 277, 278, 279, 282, 286, 293-298, 383-388, 391, 46-52, 54, 57, 73-82, 84, 85, 88 | 73 |  | 187 | 43 | (2.564, 44.057) |
| T0942 | Extend several HIS (H145, H140, H136, H246) to a pocket | 136, 137, 139, 140, 145, 196, 200, 201, 204, 246 | 10 |  | 403 | 92 | (2.992, 72.043) |
| T0947 | Compared to structure 3vyn, which reports a CYS-HIS-SER catalytic triad. Candidate SER 138 is used to define the second pocket.  Defined based on HIS166, CYS190, SER138. | 102, 133, 134, 135, 136, 137, 138, 139, 140, 162, 163, 164, 165, 166, 167, 168, 186, 187, 189, 190, 191, 192, 193,  198 | 25 |  | 438 | 97 | (5.292, 33.782) |
| T0860 | Pocket defined based on the loop: residues 33-47. Radius based method was used to extend the pocket | 128, 33, 34, 35, 36, 37, 38, 39, 40, 41, 42, 43, 44, 45, 46, 47 | 17 |  | 186 | 42 | (2.654, 54.506) |
| T0864 | Centered at chain-C residue 9-13. | 1, 2, 3, 9, 10, 11, 12, 45, 46, 47, 13 | 11 |  | 454 | 101 | (10.339, 139.28) |
| T0882 | Manually define pocket using distance cutoff based on: D1154, F1147, I1162, I1159, F1168, V1145, M1163 | 1154, 1147, 1162, 1159, 1168, 1145, 1163 | 11 |  | 518 | 116 | (2.306, 25.381) |
| T0882 | Manually define pocket using distance cutoff based on: D1154, F1147, I1162, I1159, F1168, V1145, M1163 | 1154, 1147, 1162, 1159, 1168, 1145, 1163 | 11 |  | 518 | 116 | (2.306, 25.381) |
| T0914 | T0914: fpocket 8,10,11 was merged to define the center. (radius = 10 Angstrom) | 220-229, 231, 238, 244-256, 259 | 26 |  | 444 | 97 | (13.527, 127.450) |
| T0915 | T0915: fpocket 0 was used directly to define residues, the full pocket was then extracted based on the residue list | T0915: 13, 14, 17, 18, 23, 35, 39, 53, 56, 57, 60, 63, 68 | 14 |  | 503 | 108 | (5.419, 40.854) |
| T0920-0 | Combine residues S103, A126, R241 with pocket_0 | 136, 45, 14, 135, 162, 163, 161, 46, 47, 12, 44, 9, 134, 138, 95, 210, 93, 70-73, 255, 17, 211, 187, 94, 96, 109, 110, 115 | 31 |  | 167 | 39 | (3.213, 180.131) |
| T0920-1 | Combine residues Q440, V461, E462 with pocket_6 | 382, 390, 431, 380, 456, 429, 437, 454, 430, 409, 406, 439, 428, 432 | 14 |  | 167 | 39 | (3.213, 180.131) |
| T0943-1 | Motif II: <http://www.nature.com/nsmb/journal/v9/n5/full/nsb788.html> | 98, 99, 101, 106, 107, 108, 109, 110, 111 | 9 |  | 172 | 40 | (6.497, 77.188) |
| T0943-2 | Motif II: https://www.ncbi.nlm.nih.gov/pmc/articles/PMC240672 | 128, 129, 132, 133, 134 135, | 10 |  | 172 | 40 | (6.497, 77.188) |
| T0948-0 | Centered on ARG54, Radius based method was used to redefine the pocket (radius = 9 Angstrom) | T0948 (ARG54): 47, 48, 49, 50, 51, 52, 53, 54, 55, 56, 69, 70, 74, 75, 76, 77, 78, 81 | 18 |  | 422 | 95 | (3.092, 36.111) |
| T0948-1 | Centered on SER61, Radius based method was used to redefine the pocket (radius = 10 Angstrom) | T0948 (SER61): 29, 46, 56, 57, 58, 59, 60, 61, 62, 63, 64, 65, 66, 67, 68 | 15 |  | 422 | 95 | (3.092, 36.111) |
| T0948-2 | Centered on GLY54, Radius based method was used to redefine the pocket (radius = 9 Angstrom) | T0948 (GLY54): 46, 47, 48, 49, 50, 51, 52, 53, 54, 55, 56, 57, 60, 70, 74, 75, 76 ,77, 78 | 19 |  | 410 | 93 | (3.092, 36.111) |
| T0948-3 | Centered on PRO61, Radius based method was used to redefine the pocket (radius = 10 Angstrom) | T0948 (PRO61): 29, 46, 50, 56, 57, 58, 59, 60, 61, 62, 63, 64, 65, 66, 67, 68 | 16 |  | 410 | 93 | (3.092, 36.111) |

Table S1B.

Information for the 25 targets. (1) Yellow: ligand co-crystalized in experimental structures. Holo structure; (2) Blue: known small molecule binding sites or catalytic sites. Apo structures. (3). Purple: motifs, loops and key residues involved in function. (4) Red: mutations

| Target ID | Uniprot IDs | protein/gene names | PDB codes |
| --- | --- | --- | --- |
| T0861 | P0ABK6 | Cysteine synthase A/ cysK | [5j5v](http://www.rcsb.org/pdb/cgi/explore.cgi?pdbId=5j5v) |
| T0863 | A4IGB6 | Zgc:136689/ stra6 | [5sy1](http://www.rcsb.org/pdb/cgi/explore.cgi?pdbId=5sy1) |
| T0873 | Best guess:  C6ZCR8 | UbiD-like decarboxylase/TtnD |  |
| T0879 | [C4ZEZ9](http://www.rcsb.org/pdb/search/smart.do?smartComparator=and&smartSearchSubtype_0=UpAccessionIdQuery&target=Current&accessionIdList_0=C4ZEZ9) | Peptidoglycan N-acetylglucosamine deacetylase/EUBREC_2389  “APC113058.108” was the target info from the CASP website | [5jmu](http://www.rcsb.org/pdb/cgi/explore.cgi?pdbId=5jmu) |
| T0889 | [Q89FN7](http://www.rcsb.org/pdb/search/smart.do?smartComparator=and&smartSearchSubtype_0=UpAccessionIdQuery&target=Current&accessionIdList_0=Q89FN7) | Ribitol 2-dehydrogenase / rdh | [5jo9](http://www.rcsb.org/pdb/cgi/explore.cgi?pdbId=5jo9) |
| T0891 | A0A1C7D116 | Internalin/ HYU01_03010  “Bacillus anthracis Hal NEAT domain” was the target info from the CASP website | [4ymp](http://www.rcsb.org/pdb/cgi/explore.cgi?pdbId=4ymp) |
| T0893 | [Q9X688](http://www.rcsb.org/pdb/search/smart.do?smartComparator=and&smartSearchSubtype_0=UpAccessionIdQuery&target=Current&accessionIdList_0=Q9X688) | Cell cycle histidine kinase CckA/cckA  “ccka_dhp_ca” was the target info from the CASP website | [5idj](http://www.rcsb.org/pdb/cgi/explore.cgi?pdbId=5idj) |
| T0910 |  | “PKG I” was the target info from the CASP website |  |
| T0911 |  | “D galactonate transporter” was the target info from the CASP website |  |
| T0880 |  | “C-terminal domain of MAdV2 fibre” was the target info from the CASP website |  |
| T0894 | Best guess:  A0A1S4NYE3 A0A1S4NYE4 | CDI complex from Escherichia coli STEC_O31  “CDI204-E1” was the target info from the CASP website | [5hkq](http://www.rcsb.org/pdb/cgi/explore.cgi?pdbId=5hkq) |
| T0895 | Best guess:  A0A1S4NYE3 A0A1S4NYE4 | CDI complex from Escherichia coli STEC_O31  “CDI204-E2” was the target info from the CASP website. | [5hkq](http://www.rcsb.org/pdb/cgi/explore.cgi?pdbId=5hkq) |
| T0896 | [Q6MPG8](http://www.uniprot.org/uniprot/Q6MPG8) | Uncharacterized protein/Bd0886 |  |
| T0913 | F4ZCI3 | 2-nitroimidazole nitrohydrolase/nnhA |  |
| T0917 |  | “Red Sea protein” was the target info from the CASP website. |  |
| T0942 |  | “BepA” was the target info from the CASP website. |  |
| T0947 | [Q6MQC2](http://www.uniprot.org/uniprot/Q6MQC2) | **Uncharacterized protein**/Bd0553 |  |
| T0860 | [F4MI11](http://www.rcsb.org/pdb/search/smart.do?smartComparator=and&smartSearchSubtype_0=UpAccessionIdQuery&target=Current&accessionIdList_0=F4MI11) | Fiber protein  “fibre head domain” was the target info from the CASP website | [5fjl](http://www.rcsb.org/pdb/cgi/explore.cgi?pdbId=5fjl) |
| T0864 | [O75663](http://www.rcsb.org/pdb/search/smart.do?smartComparator=and&smartSearchSubtype_0=UpAccessionIdQuery&target=Current&accessionIdList_0=O75663) | TIP41-like protein/ TIPRL | [5d9g](http://www.rcsb.org/pdb/cgi/explore.cgi?pdbId=5d9g) |
| T0882 |  | “WNK1” was the target info from the CASP website. | [5g3q](http://www.rcsb.org/pdb/cgi/explore.cgi?pdbId=5g3q) |
| T0914 |  | “CPX209” was the target info from the CASP website. |  |
| T0915 |  | “CPX209-SU2” was the target info from the CASP website. |  |
| T0920 | [C8WZ86](http://www.rcsb.org/pdb/search/smart.do?smartComparator=and&smartSearchSubtype_0=UpAccessionIdQuery&target=Current&accessionIdList_0=C8WZ86) | Extracellular ligand-binding receptor/Dret_0059  “DSM5692” was the target info from the CASP website. | [5ere](http://www.rcsb.org/pdb/cgi/explore.cgi?pdbId=5ere) |
| T0943 | [Q96PZ0](http://www.rcsb.org/pdb/search/smart.do?smartComparator=and&smartSearchSubtype_0=UpAccessionIdQuery&target=Current&accessionIdList_0=Q96PZ0) | Pseudouridylate synthase 7 homolog/ PUS7 | [5kkp](http://www.rcsb.org/pdb/cgi/explore.cgi?pdbId=5kkp) |
| T0948 | [Q8TAX9](http://www.rcsb.org/pdb/search/smart.do?smartComparator=and&smartSearchSubtype_0=UpAccessionIdQuery&target=Current&accessionIdList_0=Q8TAX9) | Gasdermin B C-terminal domain / GSDMB | [5tj4](http://www.rcsb.org/pdb/cgi/explore.cgi?pdbId=5tj4) |

Table S2A: Correlation between functional assessment and CASP assessment (all-models)

| Target | rank_cors | rmsd_cors | GDT_cors | TM_align_cors |
| --- | --- | --- | --- | --- |
| T0861 | 0.7103 | 0.4463 | 0.5640 | 0.4222 |
| T0863 | 0.5294 | 0.4614 | 0.4986 | 0.4916 |
| T0873 | 0.4768 | 0.2751 | 0.4126 | 0.3304 |
| T0879 | 0.5019 | 0.4660 | 0.5300 | 0.4127 |
| T0889 | 0.4878 | 0.4443 | 0.5007 | 0.5424 |
| T0891 | 0.4388 | 0.8815 | 0.4491 | 0.3590 |
| T0893 | 0.4489 | 0.4998 | 0.4691 | 0.5243 |
| T0910 | 0.6979 | 0.5518 | 0.7006 | 0.5787 |
| T0911 | 0.4423 | 0.2704 | 0.4383 | 0.3155 |
| *Average* | *0.5260* | *0.4774* | *0.5070* | *0.4419* |
| T0880-0 | 0.5841 | 0.4257 | 0.6235 | 0.2425 |
| T0880-1 | 0.2886 | 0.3913 | 0.4142 | 0.3652 |
| T0894 | 0.7466 | 0.8806 | 0.7702 | 0.7461 |
| T0895 | 0.5451 | 0.6015 | 0.5397 | 0.5141 |
| T0896 | 0.4436 | 0.4911 | 0.4930 | 0.4455 |
| T0913 | 0.4835 | 0.4165 | 0.4847 | 0.4587 |
| T0917 | 0.6812 | 0.7911 | 0.6949 | 0.7332 |
| T0942 | 0.4067 | 0.4420 | 0.4022 | 0.3354 |
| T0947 | 0.7191 | 0.2601 | 0.7273 | 0.7045 |
| *Average* | *0.5442* | *0.5222* | *0.5722* | *0.5050* |
| T0860 | 0.4027 | 0.6668 | 0.5125 | 0.9847 |
| T0864 | 0.6049 | 0.5891 | 0.5826 | 0.6041 |
| T0882 | 0.7321 | 0.6432 | 0.7329 | 0.7398 |
| T0914 | 0.9567 | 0.7841 | 0.9650 | 0.8392 |
| T0915 | 0.4736 | 0.4550 | 0.4660 | 0.4668 |
| T0920-0 | 0.7562 | 0.4392 | 0.7308 | 0.6220 |
| T0920-1 | 0.4436 | 0.6815 | 0.4698 | 0.4079 |
| T0943-1 | 0.5040 | 0.6113 | 0.4970 | 0.5753 |
| T0943-2 | 0.8797 | 0.8372 | 0.4439 | 0.3881 |
| *Average* | *0.6392* | *0.6342* | *0.6000* | *0.6253* |
| T0948 | 0.6249 | 0.7377 | 0.6352 | 0.7061 |
| *Average* | *0.5718* | *0.5515* | *0.5624* | *0.5305* |

Table S2B: Correlation between functional assessment and CASP assessment (model-1)

| Target | rank_cors | rmsd_cors | GDT_cors | TM_align_cors |
| --- | --- | --- | --- | --- |
| T0861 | 0.7182 | 0.6626 | 0.7142 | 0.5610 |
| T0863 | 0.5518 | 0.6555 | 0.5758 | 0.8022 |
| T0873 | 0.4861 | 0.7084 | 0.5593 | 0.5699 |
| T0879 | 0.8785 | 0.4571 | 0.8224 | 0.7699 |
| T0889 | 0.5269 | 0.9781 | 0.6359 | 0.5217 |
| T0891 | 0.5297 | 0.6087 | 0.5322 | 0.6394 |
| T0893 | 0.7801 | 0.7533 | 0.8224 | 0.8209 |
| T0910 | 0.8240 | 0.6624 | 0.8036 | 0.8125 |
| T0911 | 0.8878 | 0.9244 | 0.8475 | 0.9108 |
| *Average* | 0.6870 | 0.7123 | 0.7015 | 0.7120 |
| T0880-0 | 0.8242 | 0.8176 | 0.8217 | 0.7967 |
| T0880-1 | 0.8361 | 0.8327 | 0.8521 | 0.6990 |
| T0894 | 0.7377 | 0.8730 | 0.8281 | 0.7529 |
| T0895 | 0.6312 | 0.6551 | 0.7207 | 0.5621 |
| T0896 | 0.8253 | 0.7838 | 0.7120 | 0.6451 |
| T0913 | 0.8712 | 0.9000 | 0.8399 | 0.5407 |
| T0917 | 0.7346 | 0.9656 | 0.7664 | 0.8056 |
| T0942 | 0.9276 | 0.7841 | 0.7887 | 0.8413 |
| T0947 | 0.7203 | 0.5009 | 0.7232 | 0.6783 |
| *Average* | 0.7898 | 0.7903 | 0.7836 | 0.7024 |
| T0860 | 0.6878 | 0.8285 | 0.7188 | 0.7166 |
| T0864 | 0.6229 | 0.8351 | 0.6342 | 0.6952 |
| T0882 | 0.8510 | 0.7271 | 0.8492 | 0.8298 |
| T0914 | 0.8503 | 0.8664 | 0.8603 | 0.7990 |
| T0915 | 0.4125 | 0.5005 | 0.4786 | 0.5331 |
| T0920-0 | 0.7499 | 0.4873 | 0.7752 | 0.7864 |
| T0920-1 | 0.6219 | 0.7246 | 0.7578 | 0.7611 |
| T0943-1 | 0.7917 | 0.4780 | 0.7957 | 0.7520 |
| T0943-2 | 0.8388 | 0.6791 | 0.8666 | 0.8615 |
| *Average* | 0.7141 | 0.6807 | 0.7485 | 0.7483 |
| T0948 | 0.7380 | 0.7163 | 0.7362 | 0.7287 |
| *Average* | 0.7303 | 0.7278 | 0.7445 | 0.7209 |

Table S3. Compare GOAL and Baker-ROSETTASERVER

|  | Baker-ROSETTASERVER (server: 005,model-1) | | | | | GOAL (server: 220, model-1) | | | | |
| --- | --- | --- | --- | --- | --- | --- | --- | --- | --- | --- |
| Target ID | PF | CASP rank | RMSD | GDT | TM | PF | CASP rank | RMSD | GDT | TM |
| T0861 | -7.454* | 64 | 1.613 | 92.71 | 0.97 | -6.631 | 29 | 1.554 | 95.27 | 0.98 |
| T0863 | -2.930* | 4 | 28.084 | 9.62 | 0.26 | -1.548 | 15 | 35.222 | 9.58 | 0.31 |
| T0873 | -8.678 | 4 | 2.69 | 82.52 | 0.95 | -8.534 | 32 | 3.621 | 78.41 | 0.92 |
| T0879 | -0.702 | 28 | 5.557 | 75.11 | 0.84 | -0.602 | 49 | 6.474 | 73.07 | 0.82 |
| T0889 | -5.342* | 31 | 2.839 | 83.05 | 0.92 | -5.167* | 36 | 3.025 | 82.53 | 0.92 |
| T0891 | -3.845 | 82 | 2.953 | 86.83 | 0.89 | -4.138 | 6 | 2.69 | 90.85 | 0.92 |
| T0893 | -7.891 | 2 | 13.771 | 61.47 | 0.68 | -10.102 | 73 | 20.179 | 53.93 | 0.61 |
| T0910 | -13.69 | 9 | 3.589 | 85.3 | 0.91 | -12.103 | 31 | 3.983 | 83.51 | 0.91 |
| T0911 | -2.347 | 130 | 7.42 | 58.4 | 0.81 | -2.275 | 60 | 7.48 | 61.03 | 0.83 |
| T0880-0 | -2.536 | 144 | 21.695 | 13.6 | 0.28 | -3.006 | 267 | 20.799 | 11.53 | 0.25 |
| T0880-1 | -2.338 | 144 | 21.695 | 13.6 | 0.28 | -2.616 | 267 | 20.799 | 11.53 | 0.25 |
| T0894 | -5.870* | 28 | 5.702 | 50.87 | 0.64 | -3.487 | 204 | 16.706 | 24.3 | 0.36 |
| T0895 | -7.323 | 136 | 5.563 | 70.62 | 0.75 | -6.676 | 15 | 5.086 | 72.92 | 0.77 |
| T0896 | -2.838 | 6 | 23.127 | 24.5 | 0.39 | -2.937 | 98 | 22.983 | 21.09 | 0.36 |
| T0913 | -3.421 | 23 | 6.119 | 66.2 | 0.83 | -3.988 | 100 | 6.673 | 61.24 | 0.81 |
| T0917 | -28.11* | 5 | 3.026 | 81.64 | 0.94 | -27.83* | 9 | 3.131 | 79.22 | 0.93 |
| T0942 | -0.971 | 138 | 22.664 | 38.37 | 0.55 | -2.69 | 72 | 18.392 | 40.38 | 0.53 |
| T0947 | -6.814 | 46 | 12.2 | 61.71 | 0.7 | -8.277* | 11 | 12.678 | 64.43 | 0.7 |
| T0943-1 | -1.989 | 2 | 12.454 | 60.17 | 0.77 | -3.843* | 13 | 12.491 | 51.47 | 0.72 |
| T0943-2 | -0.947* | 2 | 12.454 | 60.17 | 0.77 | -0.588 | 13 | 12.491 | 51.47 | 0.72 |
| T0860 | -5.747* | 1 | 2.654 | 81.8 | 0.87 | -5.687* | 5 | 3.166 | 79.41 | 0.87 |
| T0882 | -5.644* | 31 | 2.69 | 87.66 | 0.84 | -5.486 | 68 | 2.973 | 85.76 | 0.87 |
| T0864 | -3.503 | 26 | 19.875 | 27.44 | 0.37 | -3.149 | 300 | 21.96 | 10.16 | 0.24 |
| T0920-0 | -8.808* | 44 | 20.447 | 42.36 | 0.59 | -7.694 | 108 | 9.589 | 36.99 | 0.6 |
| T0920-1 | -4.903 | 44 | 20.447 | 42.36 | 0.59 | -5.723* | 108 | 9.589 | 36.99 | 0.6 |
| T0914 | -7.070 | 12 | 20.778 | 17.42 | 0.3 | -8.635* | 230 | 20.494 | 11.95 | 0.31 |
| T0915 | -4.420* | 17 | 6.085 | 49.19 | 0.6 | -3.837 | 29 | 6.119 | 48.86 | 0.6 |
| T0948 | -1.299* | 102 | 4.179 | 71.48 | 0.81 | -0.511 | 25 | 4.234 | 74.5 | 0.82 |

Figure S1. Overall correlation between functional assessment and CASP official assessment (model-1). The rank is z-score combined rank, published in CASP webpage.


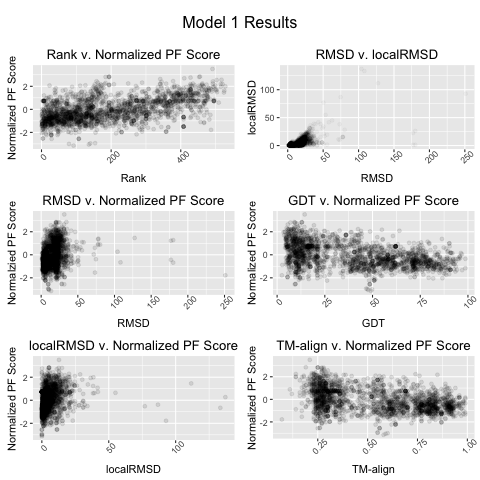


**Section 2 Assessment on three types of sites**

1. Compare performance on 6 easy targets with holo sites

T0861: LLP binding site found in T0861 that forms complex with T0862 and T0870.


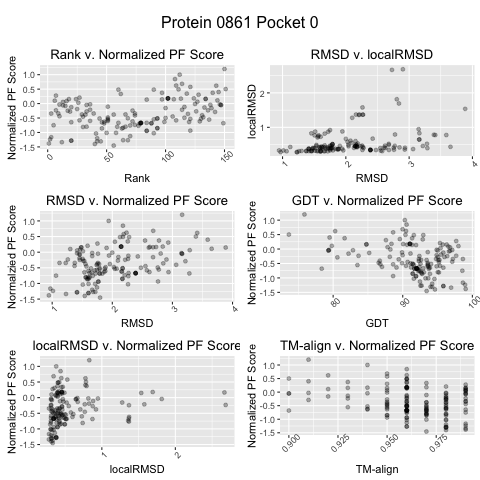

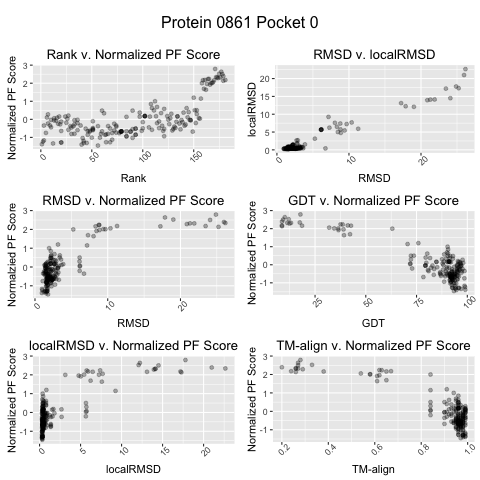


| Server ID and name | Model | PF—Zscore | GDT | CASP rank |
| --- | --- | --- | --- | --- |
| 119 HHPred0 | 1 | -1.4533 | 93.43 | 56 |
| 425 FALCON_TOPOX | 4 | -1.3986 | 94.63 | 40 |
| 275 slbio | 2 | -1.3807 | 99.04 | 1 |
| 016 FFAS-3D | 1 | -1.3361 | 96.95 | 12 |
| 425 FALCON_TOPOX | 1 | -1.3070 | 94.23 | 42 |
| 425 FALCON_TOPOX | 2 | -1.3070 | 93.99 | 45 |
| 359 Atome2_CBS | 2 | -1.2825 | 95.83 | 20 |
| 359 Atome2_CBS | 3 | -1.2825 | 95.83 | 20 |
| 405 IntFOLD4 | 2 | -1.2685 | 92.55 | 68 |
| 405 IntFOLD4 | 1 | -1.2674 | 92.71 | 64 |
| 359 Atome2_CBS | 5 | -1.2373 | 98.96 | 2 |
| 425 FALCON_TOPOX | 3 | -1.2015 | 94.23 | 42 |
| 287 MULTICOM-CLUSTER | 1 | -1.1652 | 98.64 | 3 |
| 405 IntFOLD4 | 3 | -1.1273 | 92.47 | 70 |
| 077 FALCON_TOPO | 4 | -1.0363 | 94.55 | 41 |
| 284 Seok-naive_assembly | 5 | -1.0257 | 91.59 | 90 |
| 077 FALCON_TOPO | 1 | -1.0129 | 93.67 | 50 |
| 425 FALCON_TOPOX | 5 | -1.0129 | 93.27 | 58 |
| 405 IntFOLD4 | 4 | -0.9414 | 91.91 | 84 |
| 405 IntFOLD4 | 5 | -0.9414 | 91.91 | 84 |
| 455 ACOMPMOD | 5 | -0.9369 | 88.62 | 121 |
| 005 BAKER-ROSETTASERVER | 2 | -0.8610 | 92.79 | 63 |
| 077 FALCON_TOPO | 2 | -0.8588 | 93.91 | 46 |
| 026 chuo-u2 | 2 | -0.8521 | 91.43 | 93 |
| 380 chuo-u-server | 2 | -0.8521 | 91.43 | 93 |
| 005 BAKER-ROSETTASERVER | 1 | -0.8359 | 92.71 | 64 |
| 430 GOAL_COMPLEX | 2 | -0.8097 | 95.19 | 30 |
| 275 slbio | 1 | -0.8030 | 93.03 | 59 |
| 005 BAKER-ROSETTASERVER | 4 | -0.8007 | 92.63 | 66 |
| 077 FALCON_TOPO | 3 | -0.7996 | 95.03 | 33 |

T0873: FMN binding site


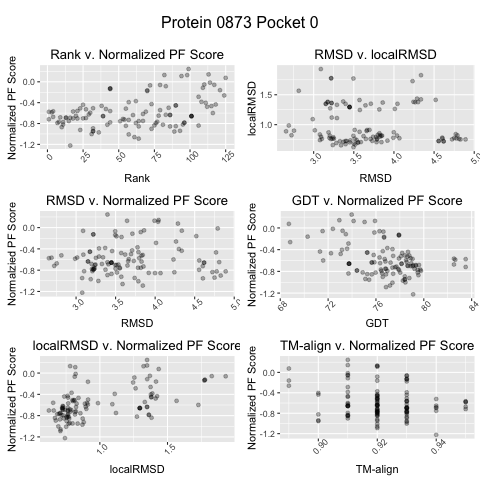

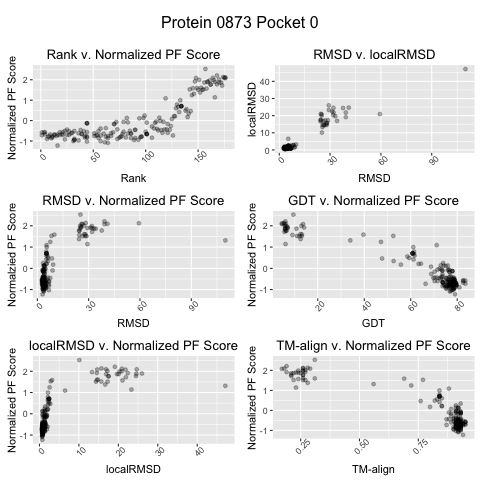


| Server ID and name | Model | PF-Zscore | GDT | CASP rank |
| --- | --- | --- | --- | --- |
| 405 IntFOLD4 | 3 | -1.2229 | 79.11 | 16 |
| 349 HHPred1 | 1 | -1.0901 | 76.84 | 64 |
| 236 MULTICOM-CONSTRUCT | 4 | -1.0606 | 77.17 | 61 |
| 250 Seok-server | 4 | -1.0234 | 77.92 | 44 |
| 275 slbio | 1 | -1.0086 | 77.38 | 57 |
| 236 MULTICOM-CONSTRUCT | 1 | -1.0025 | 78.46 | 31 |
| 407 Distill | 4 | -0.9701 | 78.25 | 36 |
| 275 slbio | 3 | -0.9541 | 75.43 | 89 |
| 446 YASARA | 3 | -0.9492 | 78.41 | 32 |
| 220 GOAL | 5 | -0.9467 | 78.41 | 32 |
| 275 slbio | 4 | -0.9398 | 75.11 | 93 |
| 220 GOAL | 3 | -0.9234 | 78.79 | 23 |
| 382 RBO_Aleph | 4 | -0.9013 | 76.57 | 73 |
| 251 myprotein-me | 1 | -0.8955 | 80.95 | 6 |
| 287 MULTICOM-CLUSTER | 5 | -0.8927 | 78.41 | 32 |
| 425 FALCON_TOPOX | 4 | -0.8701 | 79.55 | 10 |
| 345 MULTICOM-NOVEL | 4 | -0.8554 | 76.84 | 64 |
| 452 ZHOU-SPARKS-X | 2 | -0.8554 | 75.65 | 86 |
| 407 Distill | 5 | -0.8509 | 75.27 | 90 |
| 287 MULTICOM-CLUSTER | 2 | -0.8435 | 77.44 | 55 |
| 444 BhageerathH-Plus | 5 | -0.8390 | 73.7 | 106 |
| 382 RBO_Aleph | 3 | -0.8361 | 76.52 | 75 |
| 405 IntFOLD4 | 4 | -0.8230 | 78.03 | 42 |
| 028 M4T-SmotifTF | 1 | -0.8226 | 75.97 | 81 |
| 250 Seok-server | 3 | -0.8214 | 77.92 | 44 |
| 287 MULTICOM-CLUSTER | 3 | -0.8197 | 79.49 | 11 |
| 446 YASARA | 5 | -0.8189 | 74.89 | 95 |
| 382 RBO_Aleph | 5 | -0.7927 | 76.46 | 76 |
| 236 MULTICOM-CONSTRUCT | 5 | -0.7906 | 76.95 | 62 |
| 495 Seok-assembly | 1 | -0.7894 | 79.76 | 7 |

T0889: SOR binding site


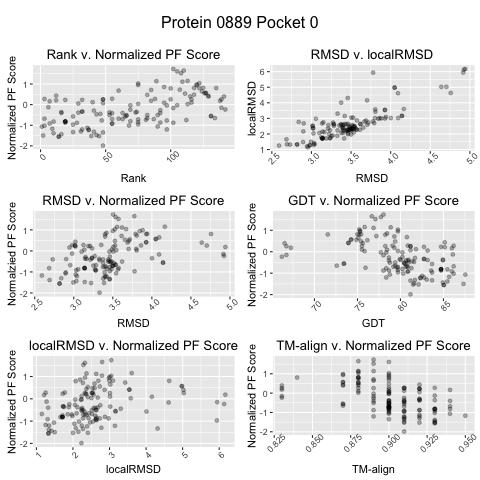

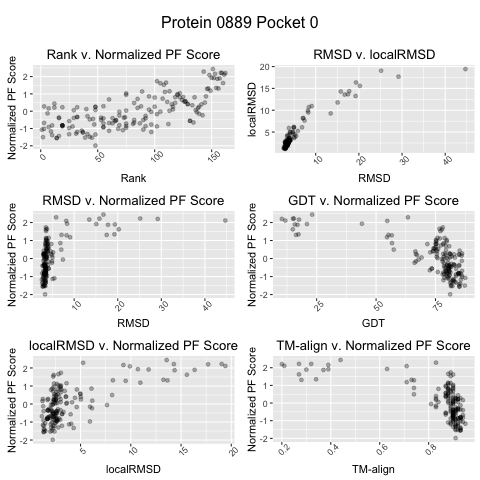


| Server ID and name | Model | PF-Zscore | GDT | CASP rank |
| --- | --- | --- | --- | --- |
| 220 GOAL | 2 | -1.9861 | 81.17 | 48 |
| 405 IntFOLD4 | 1 | -1.5517 | 85.04 | 14 |
| 405 IntFOLD4 | 2 | -1.5517 | 85.04 | 14 |
| 220 GOAL | 4 | -1.5274 | 81.48 | 45 |
| 479 Zhang-Server | 5 | -1.5244 | 82.11 | 39 |
| 405 IntFOLD4 | 5 | -1.5001 | 87.34 | 2 |
| 220 GOAL | 5 | -1.4768 | 81.59 | 43 |
| 405 IntFOLD4 | 4 | -1.4353 | 84.94 | 16 |
| 166 FFAS03 | 1 | -1.3887 | 85.46 | 10 |
| 287 MULTICOM-CLUSTER | 4 | -1.3482 | 81.17 | 48 |
| 275 slbio | 1 | -1.3320 | 83.68 | 25 |
| 220 GOAL | 3 | -1.3158 | 81.69 | 41 |
| 005 BAKER-ROSETTASERVER | 1 | -1.2986 | 83.05 | 31 |
| 236 MULTICOM-CONSTRUCT | 4 | -1.2358 | 82.32 | 37 |
| 005 BAKER-ROSETTASERVER | 3 | -1.2095 | 82.32 | 37 |
| 452 ZHOU-SPARKS-X | 3 | -1.2024 | 82.74 | 32 |
| 425 FALCON_TOPOX | 1 | -1.2014 | 78.35 | 97 |
| 183 QUARK | 1 | -1.1700 | 85.67 | 9 |
| 183 QUARK | 2 | -1.1295 | 80.75 | 60 |
| 220 GOAL | 1 | -1.1214 | 82.53 | 36 |
| 479 Zhang-Server | 2 | -1.0961 | 80.13 | 72 |
| 405 IntFOLD4 | 3 | -1.0677 | 87.55 | 1 |
| 345 MULTICOM-NOVEL | 5 | -1.0627 | 78.77 | 90 |
| 016 FFAS-3D | 1 | -1.0232 | 86.19 | 3 |
| 236 MULTICOM-CONSTRUCT | 5 | -1.0161 | 80.33 | 68 |
| 275 slbio | 4 | -0.9705 | 83.68 | 25 |
| 349 HHPred1 | 1 | -0.9574 | 79.71 | 75 |
| 048 ToyPred_email | 1 | -0.9229 | 80.86 | 55 |
| 092 RaptorX | 1 | -0.9229 | 80.86 | 55 |
| 250 Seok-server | 1 | -0.9169 | 85.98 | 7 |

T0891: HEM binding site


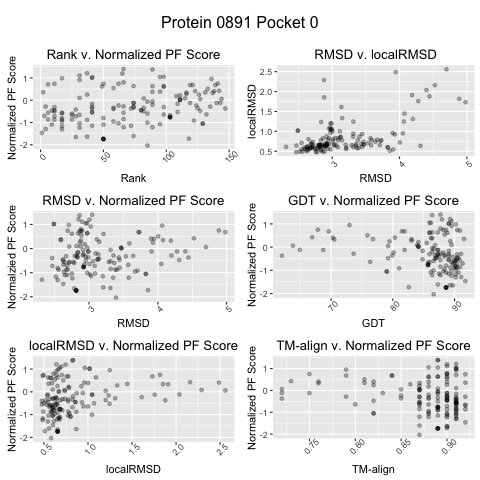

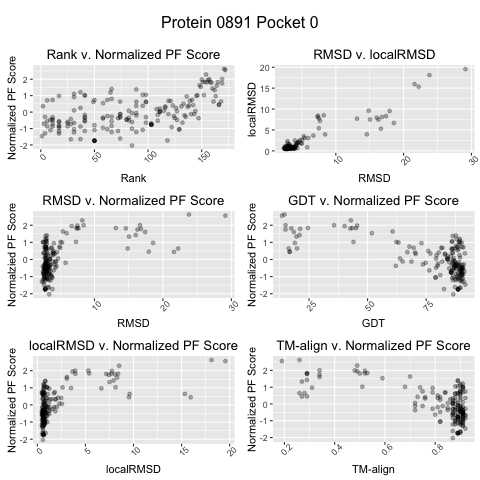


| Server ID and name | Model | PF-Zscore | GDT | CASP rank |
| --- | --- | --- | --- | --- |
| 349 HHPred1 | 1 | -2.0351 | 86.61 | 85 |
| 405 IntFOLD4 | 1 | -1.7400 | 88.62 | 50 |
| 405 IntFOLD4 | 2 | -1.7400 | 88.62 | 50 |
| 405 IntFOLD4 | 3 | -1.7400 | 88.62 | 50 |
| 405 IntFOLD4 | 4 | -1.7400 | 88.62 | 50 |
| 405 IntFOLD4 | 5 | -1.7400 | 88.62 | 50 |
| 180 PhyreTopoAlpha | 2 | -1.7263 | 82.59 | 118 |
| 236 MULTICOM-CONSTRUCT | 3 | -1.6528 | 89.73 | 28 |
| 425 FALCON_TOPOX | 3 | -1.6366 | 89.29 | 37 |
| 313 HHGG | 1 | -1.6080 | 87.28 | 74 |
| 220 GOAL | 2 | -1.4660 | 91.74 | 1 |
| 287 MULTICOM-CLUSTER | 1 | -1.3041 | 90.18 | 17 |
| 446 YASARA | 5 | -1.2917 | 91.07 | 4 |
| 345 MULTICOM-NOVEL | 4 | -1.2332 | 89.51 | 31 |
| 275 slbio | 4 | -1.2120 | 87.72 | 66 |
| 313 HHGG | 5 | -1.2095 | 86.38 | 89 |
| 236 MULTICOM-CONSTRUCT | 1 | -1.1696 | 90.18 | 17 |
| 236 MULTICOM-CONSTRUCT | 5 | -1.1472 | 87.5 | 72 |
| 425 FALCON_TOPOX | 5 | -1.1472 | 89.06 | 41 |
| 446 YASARA | 1 | -1.0962 | 88.62 | 50 |
| 236 MULTICOM-CONSTRUCT | 4 | -1.0937 | 90.62 | 9 |
| 382 RBO_Aleph | 5 | -1.0750 | 86.16 | 95 |
| 026 chuo-u2 | 5 | -1.0501 | 79.02 | 129 |
| 380 chuo-u-server | 5 | -1.0501 | 79.02 | 129 |
| 258 MUfold1 | 4 | -1.0389 | 89.51 | 31 |
| 077 FALCON_TOPO | 2 | -1.0376 | 88.17 | 62 |
| 382 RBO_Aleph | 1 | -1.0065 | 86.61 | 85 |
| 250 Seok-server | 2 | -0.9667 | 90.18 | 17 |
| 077 FALCON_TOPO | 3 | -0.9542 | 88.39 | 58 |
| 166 FFAS03 | 1 | -0.9156 | 81.03 | 122 |

T0910: ANP binding site (one subunit of a complex)


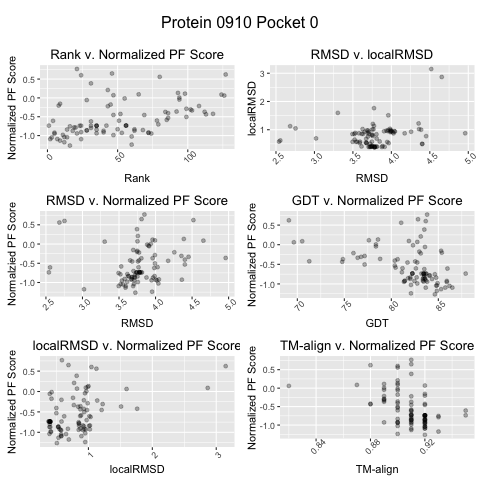

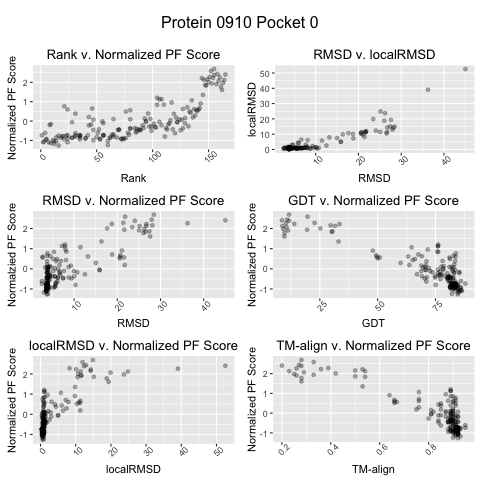


| Server ID and name | Model | PF-Zscore | GDT | CASP rank |
| --- | --- | --- | --- | --- |
| 405 IntFOLD4 | 4 | -1.2645 | 84.63 | 16 |
| 119 HHPred0 | 1 | -1.2394 | 82.09 | 60 |
| 236 MULTICOM-CONSTRUCT | 5 | -1.1764 | 84.85 | 12 |
| 005 BAKER-ROSETTASERVER | 1 | -1.1330 | 85.3 | 9 |
| 016 FFAS-3D | 1 | -1.0948 | 83.66 | 24 |
| 005 BAKER-ROSETTASERVER | 2 | -1.0935 | 86.49 | 2 |
| 005 BAKER-ROSETTASERVER | 4 | -1.0919 | 85.75 | 7 |
| 005 BAKER-ROSETTASERVER | 5 | -1.0494 | 86.19 | 3 |
| 236 MULTICOM-CONSTRUCT | 2 | -1.0436 | 84.78 | 14 |
| 236 MULTICOM-CONSTRUCT | 4 | -1.0375 | 84.85 | 12 |
| 349 HHPred1 | 1 | -1.0240 | 82.09 | 60 |
| 077 FALCON_TOPO | 3 | -0.9880 | 82.76 | 47 |
| 236 MULTICOM-CONSTRUCT | 1 | -0.9806 | 84.92 | 11 |
| 005 BAKER-ROSETTASERVER | 3 | -0.9504 | 85.9 | 5 |
| 313 HHGG | 3 | -0.9449 | 81.34 | 74 |
| 446 YASARA | 5 | -0.9433 | 83.81 | 21 |
| 236 MULTICOM-CONSTRUCT | 3 | -0.9395 | 83.43 | 35 |
| 446 YASARA | 3 | -0.9385 | 82.46 | 53 |
| 313 HHGG | 1 | -0.9285 | 81.42 | 72 |
| 446 YASARA | 4 | -0.9269 | 82.46 | 53 |
| 220 GOAL | 5 | -0.9221 | 85.9 | 5 |
| 313 HHGG | 5 | -0.9183 | 81.42 | 72 |
| 166 FFAS03 | 1 | -0.9137 | 83.36 | 40 |
| 287 MULTICOM-CLUSTER | 5 | -0.9080 | 83.66 | 24 |
| 250 Seok-server | 3 | -0.8829 | 81.34 | 74 |
| 250 Seok-server | 4 | -0.8784 | 81.27 | 76 |
| 220 GOAL | 3 | -0.8742 | 84.33 | 18 |
| 077 FALCON_TOPO | 2 | -0.8720 | 83.36 | 40 |
| 405 IntFOLD4 | 5 | -0.8697 | 82.02 | 62 |
| 048 ToyPred_email | 1 | -0.8617 | 83.58 | 28 |

T0911: GCO binding site (one subunit of a complex)


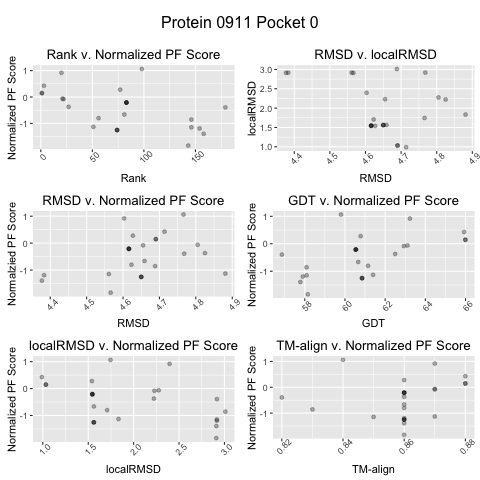

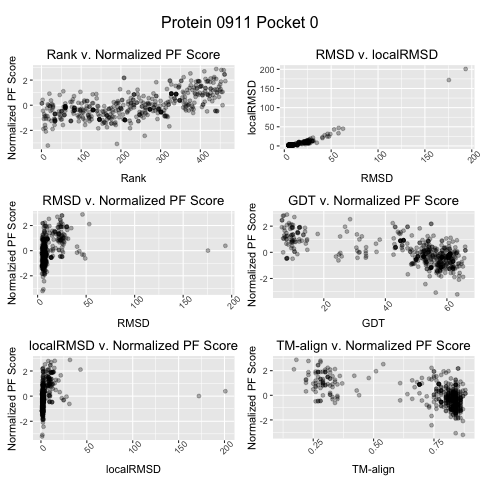


| Server ID and name | Model | PF-Zscore | GDT | CASP rank |
| --- | --- | --- | --- | --- |
| 236 MULTICOM-CONSTRUCT | 4 | -3.2110 | 63.36 | 16 |
| 479 Zhang-Server | 5 | -3.0788 | 56.37 | 190 |
| 060 KIAS-Gdansk | 4 | -2.4333 | 53.0 | 260 |
| 004 Zhang | 5 | -2.0926 | 63.91 | 11 |
| 325 wfRosetta-MUfold | 2 | -1.8962 | 62.44 | 28 |
| 220 GOAL | 4 | -1.8801 | 65.26 | 5 |
| 396 PML | 5 | -1.8400 | 58.15 | 143 |
| 357 FLOUDAS_SERVER | 1 | -1.8300 | 54.41 | 223 |
| 324 MUFOLD | 5 | -1.8120 | 63.97 | 10 |
| 243 Seok-refine | 4 | -1.7518 | 57.41 | 165 |
| 451 RaptorX-Contact | 4 | -1.6797 | 47.3 | 322 |
| 324 MUFOLD | 3 | -1.5775 | 64.15 | 9 |
| 313 HHGG | 2 | -1.5755 | 56.62 | 187 |
| 325 wfRosetta-MUfold | 3 | -1.5755 | 61.27 | 54 |
| 446 YASARA | 1 | -1.5695 | 59.44 | 110 |
| 252 wfRosetta-ProQ-ModF6 | 5 | -1.5654 | 62.07 | 33 |
| 287 MULTICOM-CLUSTER | 5 | -1.4652 | 57.9 | 154 |
| 183 QUARK | 3 | -1.4632 | 56.74 | 184 |
| 232 Chicken_George | 4 | -1.4492 | 56.92 | 176 |
| 313 HHGG | 1 | -1.4492 | 56.92 | 176 |
| 357 FLOUDAS_SERVER | 3 | -1.4472 | 54.29 | 225 |
| 243 Seok-refine | 5 | -1.4291 | 57.66 | 162 |
| 479 Zhang-Server | 2 | -1.3931 | 57.78 | 158 |
| 313 HHGG | 5 | -1.3730 | 56.68 | 186 |
| 243 Seok-refine | 3 | -1.3389 | 57.78 | 158 |
| 450 LEEab | 2 | -1.2788 | 60.11 | 94 |
| 407 Distill | 3 | -1.2748 | 58.64 | 121 |
| 079 iFold_1 | 2 | -1.2668 | 53.49 | 244 |
| 251 myprotein-me | 4 | -1.2668 | 53.49 | 244 |
| 114 Kloczkowski | 5 | -1.2568 | 62.01 | 34 |

Table S4: Servers that predict best functional relevant models (holo binding sites).

| T0861 | | T0873 | | T0889 | |
| --- | --- | --- | --- | --- | --- |
| Server /model | | Server /model | | Server /model | |
| HHPred0 | 1 | IntFOLD4 | 3 | GOAL | 2 |
| FALCON_TOPOX | 4 | HHPred1 | 1 | IntFOLD4 | 1 |
| slbio | 2 | MULTICOM-CONSTRUCT | 4 | IntFOLD4 | 2 |
| FFAS-3D | 1 | Seok-server | 4 | GOAL | 4 |
| FALCON_TOPOX | 1 | slbio | 1 | Zhang-Server | 5 |
| FALCON_TOPOX | 2 | MULTICOM-CONSTRUCT | 1 | IntFOLD4 | 5 |
| Atome2_CBS | 2 | Distill | 4 | GOAL | 5 |
| Atome2_CBS | 3 | slbio | 3 | IntFOLD4 | 4 |
| ntFOLD4 | 2 | YASARA | 3 | FFAS03 | 1 |
| IntFOLD4 | 1 | GOAL | 5 | MULTICOM-CLUSTER | 4 |
| Atome2_CBS | 5 | slbio | 4 | slbio | 1 |
| FALCON_TOPOX | 3 | GOAL | 3 | GOAL | 3 |
| MULTICOM-CLUSTER | 1 | RBO_Aleph | 4 | BAKER-ROSETTASERVER | 1 |
| IntFOLD4 | 3 | myprotein-me | 1 | MULTICOM-CONSTRUCT | 4 |
| FALCON_TOPO | 4 | MULTICOM-CLUSTER | 5 | BAKER-ROSETTASERVER | 3 |
| Seok-naive_assembly | 5 | FALCON_TOPOX | 4 | ZHOU-SPARKS-X | 3 |
| FALCON_TOPO | 1 | MULTICOM-NOVEL | 4 | FALCON_TOPOX | 1 |
| FALCON_TOPOX | 5 | ZHOU-SPARKS-X | 2 | QUARK | 1 |
| IntFOLD4 | 4 | Distill | 5 | QUARK | 2 |
| IntFOLD4 | 5 | MULTICOM-CLUSTER | 2 | GOAL | 1 |
| ACOMPMOD | 5 | BhageerathH-Plus | 5 | Zhang-Server | 2 |
| BAKER-ROSETTASERVER | 2 | RBO_Aleph | 3 | IntFOLD4 | 3 |
| FALCON_TOPO | 2 | IntFOLD4 | 4 | MULTICOM-NOVEL | 5 |
| chuo-u2 | 2 | M4T-SmotifTF | 1 | FFAS-3D | 1 |
| chuo-u-server | 2 | Seok-server | 3 | MULTICOM-CONSTRUCT | 5 |
| BAKER-ROSETTASERVER | 1 | MULTICOM-CLUSTER | 3 | slbio | 4 |
| GOAL_COMPLEX | 2 | YASARA | 5 | 3HHPred1 | 1 |
| slbio | 1 | RBO_Aleph | 5 | ToyPred_email | 1 |
| BAKER-ROSETTASERVER | 4 | MULTICOM-CONSTRUCT | 5 | RaptorX | 1 |
| FALCON_TOPO | 3 | Seok-assembly | 1 | Seok-server | 1 |

| T0891 | | T0910 | | T0911 | |
| --- | --- | --- | --- | --- | --- |
| Server /model | | Server /model | | Server /model | |
| HHPred1 | 1 | IntFOLD4 | 4 | MULTICOM-CONSTRUCT | 4 |
| IntFOLD4 | 1 | HHPred0 | 1 | Zhang-Server | 5 |
| IntFOLD4 | 2 | MULTICOM-CONSTRUCT | 5 | KIAS-Gdansk | 4 |
| IntFOLD4 | 3 | BAKER-ROSETTASERVER | 1 | Zhang | 5 |
| IntFOLD4 | 4 | FFAS-3D | 1 | wfRosetta-MUfold | 2 |
| IntFOLD4 | 5 | BAKER-ROSETTASERVER | 2 | GOAL | 4 |
| PhyreTopoAlpha | 2 | BAKER-ROSETTASERVER | 4 | PML | 5 |
| MULTICOM-CONSTRUCT | 3 | BAKER-ROSETTASERVER | 5 | FLOUDAS_SERVER | 1 |
| FALCON_TOPOX | 3 | MULTICOM-CONSTRUCT | 2 | MUFOLD | 5 |
| HHGG | 1 | MULTICOM-CONSTRUCT | 4 | Seok-refine | 4 |
| GOAL | 2 | HHPred1 | 1 | RaptorX-Contact | 4 |
| MULTICOM-CLUSTER | 1 | FALCON_TOPO | 3 | MUFOLD | 3 |
| YASARA | 5 | MULTICOM-CONSTRUCT | 1 | HHGG | 2 |
| MULTICOM-NOVEL | 4 | BAKER-ROSETTASERVER | 3 | wfRosetta-MUfold | 3 |
| slbio | 4 | HHGG | 3 | YASARA | 1 |
| HHGG | 5 | YASARA | 5 | wfRosetta-ProQ-ModF6 | 5 |
| MULTICOM-CONSTRUCT | 1 | MULTICOM-CONSTRUCT | 3 | MULTICOM-CLUSTER | 5 |
| MULTICOM-CONSTRUCT | 5 | YASARA | 3 | QUARK | 3 |
| FALCON_TOPOX | 5 | HHGG | 1 | Chicken_George | 4 |
| YASARA | 1 | YASARA | 4 | HHGG | 1 |
| MULTICOM-CONSTRUCT | 4 | GOAL | 5 | FLOUDAS_SERVER | 3 |
| RBO_Aleph | 5 | HHGG | 5 | Seok-refine | 5 |
| chuo-u2 | 5 | FFAS03 | 1 | Zhang-Server | 2 |
| chuo-u-server | 5 | MULTICOM-CLUSTER | 5 | HHGG | 5 |
| MUfold1 | 4 | Seok-server | 3 | Seok-refine | 3 |
| FALCON_TOPO | 2 | Seok-server | 4 | LEEab | 2 |
| RBO_Aleph | 1 | GOAL | 3 | Distill | 3 |
| Seok-server | 2 | FALCON_TOPO | 2 | iFold_1 | 2 |
| FALCON_TOPO | 3 | IntFOLD4 | 5 | myprotein-me | 4 |
| FFAS03 | 1 | ToyPred_email | 1 | Kloczkowski | 5 |

2. Compare performance on 6 easy targets with apo sites

T0942


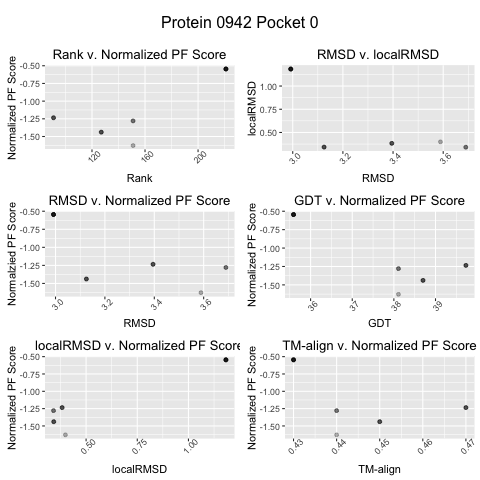

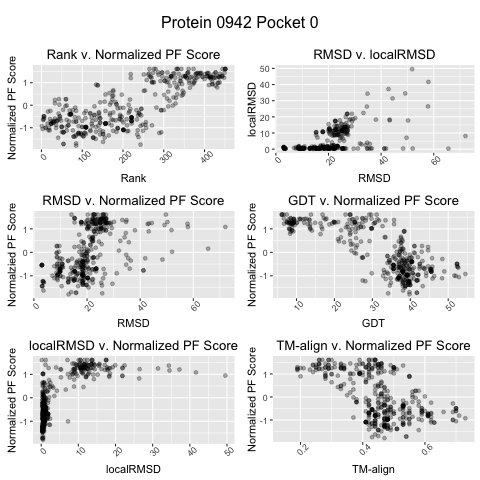


| Server ID and name | Model | PF—Zscore | GDT | CASP rank |
| --- | --- | --- | --- | --- |
| 303 wfMESHI-TIGRESS | 4 | -1.7898 | 39.79 | 85 |
| 382      RBO_Aleph | 5 | -1.7473 | 34.17 | 243 |
| 405      IntFOLD4 | 3 | -1.7212 | 41.73 | 54 |
| 320      raghavagps | 3 | -1.7212 | 41.73 | 54 |
| 411      Pcomb-domain | 4 | -1.6958 | 39.79 | 85 |
| 042      Elofsson | 3 | -1.6280 | 38.11 | 151 |
| 016      FFAS-3D | 1 | -1.4968 | 38.57 | 133 |
| 073      Wallner | 1 | -1.4431 | 43.28 | 41 |
| 236      MULTICOM-CONSTRUCT | 1 | -1.4416 | 38.05 | 155 |
| 067      wfRstta-PQ2-Seder | 1 | -1.4386 | 38.7 | 127 |
| 028      M4T-SmotifTF | 1 | -1.4386 | 38.7 | 127 |
| 079      iFold_1 | 1 | -1.4386 | 38.7 | 127 |
| 382      RBO_Aleph | 1 | -1.4140 | 34.62 | 238 |
| 464      tsspred2 | 1 | -1.3879 | 39.99 | 79 |
| 486      TASSER | 5 | -1.3879 | 39.99 | 79 |
| 464      tsspred2 | 4 | -1.3857 | 39.86 | 83 |
| 239      wfAll-Cheng | 5 | -1.3782 | 39.41 | 103 |
| 303      wfMESHI-TIGRESS | 2 | -1.3782 | 39.41 | 103 |
| 464      tsspred2 | 3 | -1.3395 | 37.92 | 165 |
| 243      Seok-refine | 4 | -1.3245 | 39.53 | 99 |
| 411      Pcomb-domain | 5 | -1.3163 | 39.79 | 85 |
| 405      IntFOLD4 | 4 | -1.2977 | 38.18 | 144 |
| 382      RBO_Aleph | 3 | -1.2940 | 34.88 | 235 |
| 396 PML | 1 | -1.2820 | 39.73 | 91 |
| 079      iFold_1 | 3 | -1.2791 | 38.11 | 151 |
| 166      FFAS03 | 1 | -1.2791 | 38.11 | 151 |
| 243      Seok-refine | 2 | -1.2761 | 39.15 | 109 |
| 102      Kiharalab | 4 | -1.2560 | 39.6 | 97 |
| 479      Zhang-Server | 1 | -1.2560 | 39.6 | 97 |
| 067      wfRstta-PQ2-Seder | 2 | -1.2351 | 39.73 | 91 |

T0894


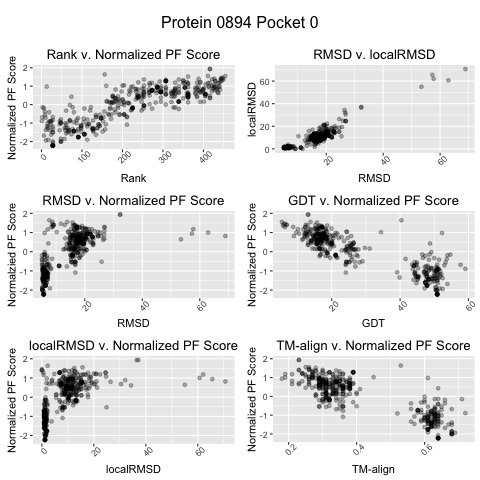


| Server ID and name | Model | PF—Zscore | GDT | CASP rank |
| --- | --- | --- | --- | --- |
| 247      BAKER | 1 | -2.2214 | 50.87 | 28 |
| 203      ProQ2 | 1 | -2.2214 | 50.87 | 28 |
| 486      TASSER | 2 | -2.2214 | 50.87 | 28 |
| 417      VoroMQA-select | 4 | -2.2214 | 50.87 | 28 |
| 232      Chicken_George | 3 | -2.2214 | 50.87 | 28 |
| 114      Kloczkowski | 1 | -2.2214 | 50.87 | 28 |
| 363      Faraggi | 2 | -2.2214 | 50.87 | 28 |
| 067      wfRstta-PQ2-Seder | 2 | -2.2214 | 50.87 | 28 |
| 005      BAKER-ROSETTASERVER | 1 | -2.2214 | 50.87 | 28 |
| 486      TASSER | 1 | -2.0656 | 50.87 | 28 |
| 203      ProQ2 | 4 | -2.0047 | 50.52 | 48 |
| 220      GOAL | 2 | -2.0047 | 50.52 | 48 |
| 102      Kiharalab | 5 | -2.0047 | 50.52 | 48 |
| 243      Seok-refine | 2 | -1.9948 | 50.87 | 28 |
| 243      Seok-refine | 5 | -1.9402 | 52.45 | 11 |
| 243      Seok-refine | 4 | -1.9126 | 51.57 | 17 |
| 456      wfRosetta-Wallner | 2 | -1.9091 | 47.2 | 113 |
| 439      MULTICOM | 5 | -1.8616 | 50.35 | 57 |
| 243      Seok-refine | 3 | -1.8489 | 50.52 | 48 |
| 005      BAKER-ROSETTASERVER | 2 | -1.7611 | 47.9 | 91 |
| 247      BAKER | 2 | -1.7611 | 47.9 | 91 |
| 417      VoroMQA-select | 5 | -1.7611 | 47.9 | 91 |
| 203      ProQ2 | 3 | -1.7611 | 47.9 | 91 |
| 067      wfRstta-PQ2-Seder | 5 | -1.7611 | 47.9 | 91 |
| 363      Faraggi | 1 | -1.7611 | 47.9 | 91 |
| 073      Wallner | 1 | -1.6818 | 53.85 | 6 |
| 411      Pcomb-domain | 3 | -1.6364 | 51.05 | 24 |
| 077      FALCON_TOPO | 2 | -1.6350 | 44.76 | 135 |
| 073      Wallner | 3 | -1.6088 | 48.78 | 74 |
| 005      BAKER-ROSETTASERVER | 4 | -1.6074 | 47.38 | 106 |

T0895


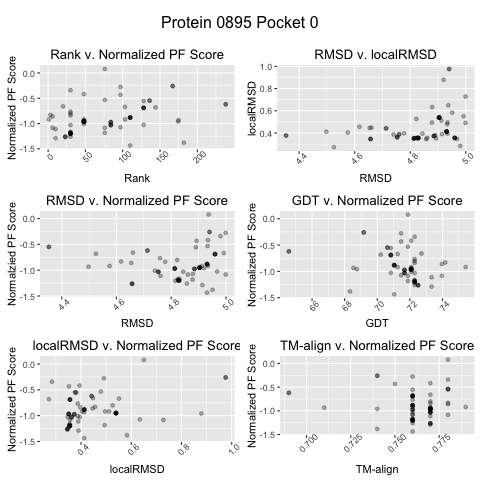

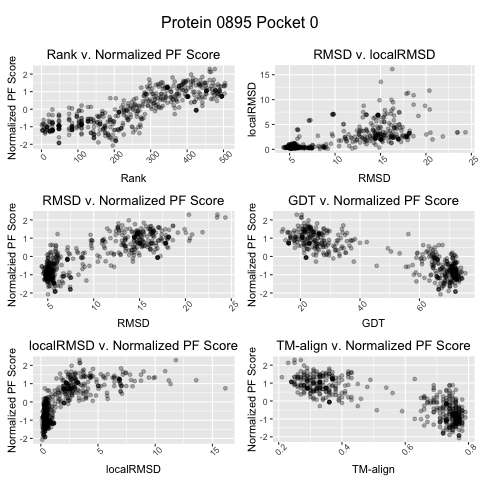


| Server ID and name | Model | PF—Zscore | GDT | CASP rank |
| --- | --- | --- | --- | --- |
| 456      wfRosetta-Wallner | 5 | -2.0728 | 67.08 | 196 |
| 114      Kloczkowski | 1 | -1.9238 | 72.08 | 48 |
| 005      BAKER-ROSETTASERVER | 3 | -1.9238 | 72.08 | 48 |
| 067      wfRstta-PQ2-Seder | 1 | -1.9238 | 72.08 | 48 |
| 247      BAKER | 4 | -1.8940 | 69.79 | 156 |
| 005      BAKER-ROSETTASERVER | 4 | -1.8031 | 66.46 | 204 |
| 067      wfRstta-PQ2-Seder | 4 | -1.8031 | 66.46 | 204 |
| 417      VoroMQA-select | 5 | -1.8031 | 66.46 | 204 |
| 247      BAKER | 2 | -1.7278 | 67.5 | 194 |
| 498      AP_1 | 4 | -1.6620 | 66.46 | 204 |
| 252      wfRosetta-ProQ-ModF6 | 4 | -1.6594 | 70.21 | 144 |
| 456      wfRosetta-Wallner | 2 | -1.6270 | 67.71 | 189 |
| 252      wfRosetta-ProQ-ModF6 | 2 | -1.6207 | 70.21 | 144 |
| 456      wfRosetta-Wallner | 3 | -1.6207 | 70.21 | 144 |
| 498      AP_1 | 3 | -1.6040 | 72.08 | 48 |
| 247      BAKER | 1 | -1.5961 | 68.12 | 184 |
| 247      BAKER | 3 | -1.5109 | 68.54 | 175 |
| 446      YASARA | 1 | -1.4717 | 68.12 | 184 |
| 363      Faraggi | 5 | -1.4717 | 68.12 | 184 |
| 393      MESHI | 3 | -1.4670 | 71.88 | 76 |
| 252 wfRosetta-ProQ-ModF6 | 1 | -1.4649 | 67.08 | 196 |
| 252 wfRosetta-ProQ-ModF6 | 3 | -1.4487 | 65.21 | 226 |
| 089 Pcons | 5 | -1.4357 | 71.04 | 110 |
| 417 VoroMQA-select | 1 | -1.4320 | 70.21 | 144 |
| 220 GOAL | 5 | -1.4320 | 70.21 | 144 |
| 102 Kiharalab | 2 | -1.4320 | 70.21 | 144 |
| 324 MUFOLD | 2 | -1.4221 | 69.58 | 157 |
| 393      MESHI | 4 | -1.3902 | 73.33 | 10 |
| 064 Jones-UCL | 1 | -1.3844 | 71.04 | 110 |
| 411      Pcomb-domain | 1 | -1.3797 | 68.33 | 182 |

T0896


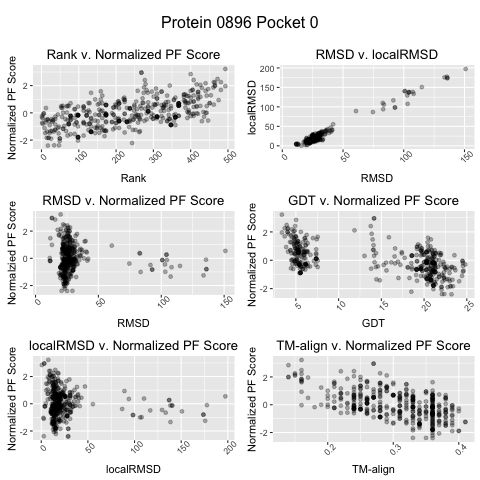


| Server ID and name | Model | PF—Zscore | GDT | CASP rank |
| --- | --- | --- | --- | --- |
| 439      MULTICOM | 5 | -2.4031 | 23.21 | 17 |
| 252      wfRosetta-ProQ-ModF6 | 4 | -2.3879 | 22.09 | 47 |
| 446      YASARA | 5 | -2.3841 | 22.37 | 34 |
| 073      Wallner | 4 | -2.2184 | 18.51 | 243 |
| 425      FALCON_TOPOX | 2 | -2.0641 | 22.04 | 49 |
| 092      RaptorX | 1 | -2.0375 | 21.09 | 98 |
| 077      FALCON_TOPO | 1 | -1.9009 | 22.76 | 26 |
| 005      BAKER-ROSETTASERVER | 5 | -1.8820 | 22.04 | 49 |
| 247      BAKER | 5 | -1.8820 | 22.04 | 49 |
| 011      LEE | 5 | -1.8390 | 23.38 | 14 |
| 005      BAKER-ROSETTASERVER | 4 | -1.8364 | 23.1 | 19 |
| 247      BAKER | 4 | -1.8364 | 23.1 | 19 |
| 079      iFold_1 | 4 | -1.7846 | 21.09 | 98 |
| 232      Chicken_George | 4 | -1.7846 | 21.09 | 98 |
| 232 Chicken_George | 3 | -1.7846 | 21.09 | 98 |
| 048 ToyPred_email | 1 | -1.7846 | 21.09 | 98 |
| 342 ToyPred | 1 | -1.7846 | 21.09 | 98 |
| 393 MESHI | 4 | -1.7112 | 20.36 | 169 |
| 450 LEEab | 3 | -1.6695 | 21.03 | 113 |
| 456 wfRosetta-Wallner | 2 | -1.6669 | 21.36 | 70 |
| 456 wfRosetta-Wallner | 5 | -1.5708 | 21.25 | 78 |
| 450 LEEab | 1 | -1.5468 | 21.2 | 89 |
| 456 wfRosetta-Wallner | 3 | -1.5076 | 22.15 | 46 |
| 017 McGuffin | 1 | -1.4899 | 20.75 | 144 |
| 011 LEE | 1 | -1.4810 | 24.11 | 10 |
| 077      FALCON_TOPO | 4 | -1.4747 | 22.99 | 22 |
| 411      Pcomb-domain | 4 | -1.4671 | 20.53 | 155 |
| 384 wfMESHI-Seok | 3 | -1.4431 | 19.18 | 223 |
| 073      Wallner | 3 | -1.4152 | 14.82 | 262 |
| 417 VoroMQA-select | 5 | -1.3862 | 20.8 | 133 |

T0913


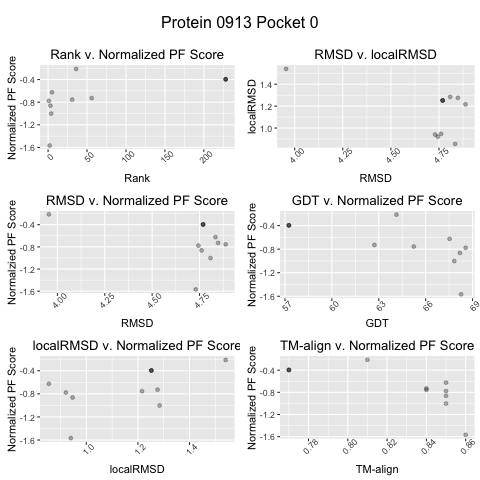

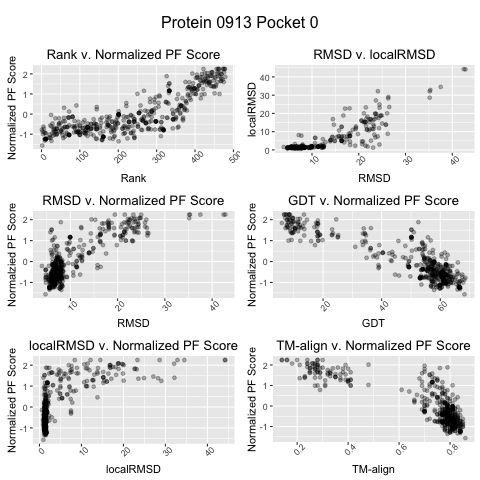


| Server ID and name | Model | PF—Zscore | GDT | CASP rank |
| --- | --- | --- | --- | --- |
| 450 LEEab | 5 | -1.5669 | 68.27 | 2 |
| 324 MUFOLD | 3 | -1.3556 | 57.91 | 201 |
| 324 MUFOLD | 1 | -1.3326 | 65.9 | 26 |
| 001 Bates_BMM | 5 | -1.3227 | 66.79 | 9 |
| 420 KF-Consensus | 1 | -1.2866 | 59.91 | 158 |
| 349 HHPred1 | 1 | -1.2866 | 59.91 | 158 |
| 119 HHPred0 | 1 | -1.2783 | 59.91 | 158 |
| 011 LEE | 3 | -1.2422 | 66.86 | 8 |
| 005 BAKER-ROSETTASERVER | 2 | -1.2405 | 66.57 | 11 |
| 303 wfMESHI-TIGRESS | 1 | -1.2405 | 66.57 | 11 |
| 498 AP_1 | 1 | -1.2405 | 66.57 | 11 |
| 207 rluethy | 1 | -1.2405 | 66.57 | 11 |
| 203 ProQ2 | 5 | -1.2405 | 66.57 | 11 |
| 417 VoroMQA-select | 3 | -1.2405 | 66.57 | 11 |
| 102 Kiharalab | 2 | -1.2405 | 66.57 | 11 |
| 247 BAKER | 3 | -1.2405 | 66.57 | 11 |
| 252 wfRosetta-ProQ-ModF6 | 3 | -1.2331 | 60.43 | 144 |
| 324 MUFOLD | 4 | -1.2298 | 58.65 | 186 |
| 384 wfMESHI-Seok | 2 | -1.2216 | 60.5 | 142 |
| 239 wfAll-Cheng | 3 | -1.2109 | 57.4 | 224 |
| 077      FALCON_TOPO | 1 | -1.2060 | 59.17 | 178 |
| 064 Jones-UCL | 1 | -1.1945 | 66.12 | 24 |
| 060 KIAS-Gdansk | 5 | -1.1945 | 62.65 | 57 |
| 464      tsspred2 | 3 | -1.1838 | 56.88 | 242 |
| 464      tsspred2 | 1 | -1.1838 | 56.88 | 242 |
| 011 LEE | 2 | -1.1813 | 66.35 | 21 |
| 393      MESHI | 2 | -1.1517 | 66.49 | 20 |
| 498      AP_1 | 4 | -1.1361 | 59.84 | 161 |
| 220 GOAL | 3 | -1.1287 | 59.84 | 161 |
| 303 wfMESHI-TIGRESS | 4 | -1.1287 | 59.84 | 161 |

T0917


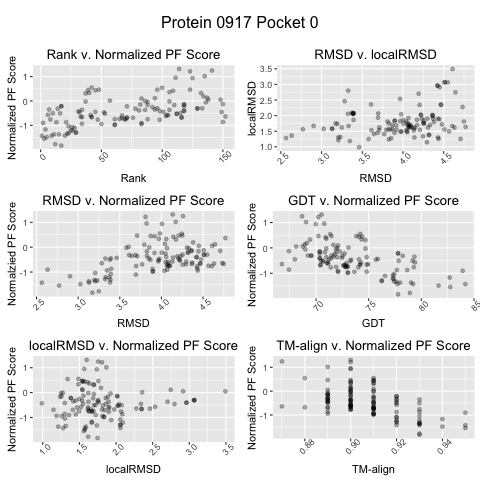

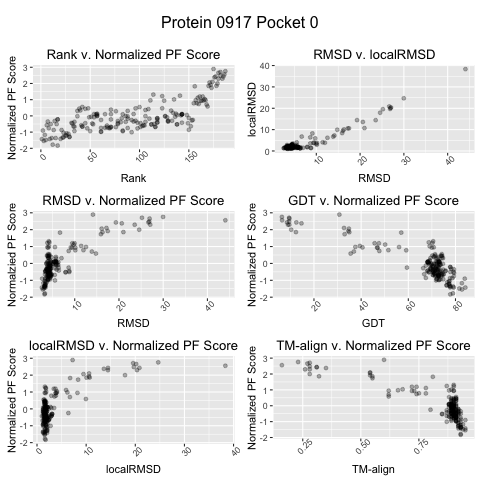


| Server ID and name | Model | PF—Zscore | GDT | CASP rank |
| --- | --- | --- | --- | --- |
| 220 GOAL | 4 | -1.8306 | 77.79 | 17 |
| 220 GOAL | 2 | -1.7745 | 78.91 | 12 |
| 005 BAKER-ROSETTASERVER | 3 | -1.5425 | 82.75 | 3 |
| 005 BAKER-ROSETTASERVER | 1 | -1.4856 | 81.64 | 5 |
| 005 BAKER-ROSETTASERVER | 5 | -1.4292 | 84.18 | 1 |
| 220 GOAL | 1 | -1.4277 | 79.22 | 9 |
| 313 HHGG | 3 | -1.4205 | 77.73 | 20 |
| 220 GOAL | 3 | -1.3694 | 77.98 | 15 |
| 430 GOAL_COMPLEX | 2 | -1.3688 | 79.03 | 11 |
| 430 GOAL_COMPLEX | 4 | -1.3386 | 77.61 | 22 |
| 430 GOAL_COMPLEX | 5 | -1.3211 | 77.42 | 23 |
| 349 HHPred1 | 1 | -1.3042 | 76.24 | 29 |
| 119 HHPred0 | 1 | -1.2863 | 76.24 | 29 |
| 313 HHGG | 1 | -1.2107 | 77.36 | 25 |
| 430 GOAL_COMPLEX | 3 | -1.2088 | 77.3 | 26 |
| 005 BAKER-ROSETTASERVER | 4 | -1.1697 | 82.69 | 4 |
| 446 YASARA | 2 | -1.1026 | 76.55 | 28 |
| 313 HHGG | 2 | -1.0758 | 77.3 | 26 |
| 220 GOAL | 5 | -1.0395 | 77.42 | 23 |
| 446 YASARA | 5 | -1.0060 | 71.77 | 92 |
| 236      MULTICOM-CONSTRUCT | 2 | -0.9768 | 73.02 | 61 |
| 236      MULTICOM-CONSTRUCT | 3 | -0.9768 | 73.02 | 61 |
| 345      MULTICOM-NOVEL | 2 | -0.9466 | 72.08 | 83 |
| 444 BhageerathH-Plus | 5 | -0.9437 | 73.64 | 50 |
| 430 GOAL_COMPLEX | 1 | -0.9364 | 74.81 | 34 |
| 236      MULTICOM-CONSTRUCT | 4 | -0.9054 | 72.08 | 83 |
| 005 BAKER-ROSETTASERVER | 2 | -0.9033 | 83.93 | 2 |
| 236      MULTICOM-CONSTRUCT | 5 | -0.8731 | 78.66 | 13 |
| 313 HHGG | 4 | -0.8719 | 77.67 | 21 |
| 467 Pareto-server | 2 | -0.8562 | 67.43 | 150 |

Table S5: Servers that predict best functional relevant models (apo binding sites).

| T0942 | | T0894 | | T0895 | |
| --- | --- | --- | --- | --- | --- |
| Server /model | | Server /model | | Server /model | |
| wfMESHI-TIGRESS | 4 | BAKER | 1 | wfRosetta-Wallner | 5 |
| RBO_Aleph | 5 | ProQ2 | 1 | Kloczkowski | 1 |
| IntFOLD4 | 3 | TASSER | 2 | BAKER-ROSETTASERVER | 3 |
| raghavagps | 3 | VoroMQA-select | 4 | wfRstta-PQ2-Seder | 1 |
| Pcomb-domain | 4 | Chicken_George | 3 | BAKER | 4 |
| Elofsson | 3 | Kloczkowski | 1 | BAKER-ROSETTASERVER | 4 |
| FFAS-3D | 1 | 3Faraggi | 2 | wfRstta-PQ2-Seder | 4 |
| Wallner | 1 | wfRstta-PQ2-Seder | 2 | VoroMQA-select | 5 |
| MULTICOM-CONSTRUCT | 1 | BAKER-ROSETTASERVER | 1 | BAKER | 2 |
| wfRstta-PQ2-Seder | 1 | TASSER | 1 | AP_1 | 4 |
| M4T-SmotifTF | 1 | ProQ2 | 4 | wfRosetta-ProQ-ModF6 | 4 |
| iFold_1 | 1 | GOAL | 2 | wfRosetta-Wallner | 2 |
| RBO_Aleph | 1 | Kiharalab | 5 | wfRosetta-ProQ-ModF6 | 2 |
| tsspred2 | 1 | Seok-refine | 2 | wfRosetta-Wallner | 3 |
| TASSER | 5 | Seok-refine | 5 | AP_1 | 3 |
| tsspred2 | 4 | Seok-refine | 4 | BAKER | 1 |
| wfAll-Cheng | 5 | wfRosetta-Wallner | 2 | BAKER | 3 |
| wfMESHI-TIGRESS | 2 | MULTICOM | 5 | YASARA | 1 |
| tsspred2 | 3 | Seok-refine | 3 | Faraggi | 5 |
| Seok-refine | 4 | BAKER-ROSETTASERVER | 2 | MESHI | 3 |
| Pcomb-domain | 5 | BAKER | 2 | wfRosetta-ProQ-ModF6 | 1 |
| IntFOLD4 | 4 | VoroMQA-select | 5 | wfRosetta-ProQ-ModF6 | 3 |
| RBO_Aleph | 3 | ProQ2 | 3 | Pcons | 5 |
| PML | 1 | wfRstta-PQ2-Seder | 5 | VoroMQA-select | 1 |
| iFold_1 | 3 | Faraggi | 1 | GOAL | 5 |
| FFAS03 | 1 | Wallner | 1 | Kiharalab | 2 |
| Seok-refine | 2 | Pcomb-domain | 3 | MUFOLD | 2 |
| Kiharalab | 4 | FALCON_TOPO | 2 | MESHI | 4 |
| Zhang-Server | 1 | Wallner | 3 | Jones-UCL | 1 |
| wfRstta-PQ2-Seder | 2 | BAKER-ROSETTASERVER | 4 | Pcomb-domain | 1 |

| T0896 | | T0913 | | T0917 | |
| --- | --- | --- | --- | --- | --- |
| Server /model | | Server /model | | Server /model | |
| MULTICOM | 5 | LEEab | 5 | GOAL | 4 |
| wfRosetta-ProQ-ModF6 | 4 | MUFOLD | 3 | GOAL | 2 |
| YASARA | 5 | MUFOLD | 1 | BAKER-ROSETTASERVER | 3 |
| Wallner | 4 | Bates_BMM | 5 | BAKER-ROSETTASERVER | 1 |
| FALCON_TOPOX | 2 | KF-Consensus | 1 | BAKER-ROSETTASERVER | 5 |
| RaptorX | 1 | HHPred1 | 1 | GOAL | 1 |
| FALCON_TOPO | 1 | HHPred0 | 1 | HHGG | 3 |
| BAKER-ROSETTASERVER | 5 | LEE | 3 | GOAL | 3 |
| BAKER | 5 | BAKER-ROSETTASERVER | 2 | GOAL_COMPLEX | 2 |
| LEE | 5 | wfMESHI-TIGRESS | 1 | GOAL_COMPLEX | 4 |
| BAKER-ROSETTASERVER | 4 | AP_1 | 1 | GOAL_COMPLEX | 5 |
| BAKER | 4 | rluethy | 1 | HHPred1 | 1 |
| iFold_1 | 4 | ProQ2 | 5 | HHPred0 | 1 |
| Chicken_George | 4 | VoroMQA-select | 3 | HHGG | 1 |
| Chicken_George | 3 | Kiharalab | 2 | GOAL_COMPLEX | 3 |
| ToyPred_email | 1 | BAKER | 3 | BAKER-ROSETTASERVER | 4 |
| ToyPred | 1 | wfRosetta-ProQ-ModF6 | 3 | YASARA | 2 |
| MESHI | 4 | MUFOLD | 4 | HHGG | 2 |
| LEEab | 3 | wfMESHI-Seok | 2 | GOAL | 5 |
| wfRosetta-Wallner | 2 | wfAll-Cheng | 3 | YASARA | 5 |
| wfRosetta-Wallner | 5 | FALCON_TOPO | 1 | MULTICOM-CONSTRUCT | 2 |
| LEEab | 1 | Jones-UCL | 1 | MULTICOM-CONSTRUCT | 3 |
| wfRosetta-Wallner | 3 | KIAS-Gdansk | 5 | MULTICOM-NOVEL | 2 |
| McGuffin | 1 | tsspred2 | 3 | BhageerathH-Plus | 5 |
| LEE | 1 | tsspred2 | 1 | GOAL_COMPLEX | 1 |
| FALCON_TOPO | 4 | LEE | 2 | MULTICOM-CONSTRUCT | 4 |
| Pcomb-domain | 4 | MESHI | 2 | BAKER-ROSETTASERVER | 2 |
| wfMESHI-Seok | 3 | AP_1 | 4 | MULTICOM-CONSTRUCT | 5 |
| Wallner | 3 | GOAL | 3 | HHGG | 4 |
| VoroMQA-select | 5 | wfMESHI-TIGRESS | 4 | Pareto-server | 2 |

3. Compare performance on 4 easy targets with key patches

T0860


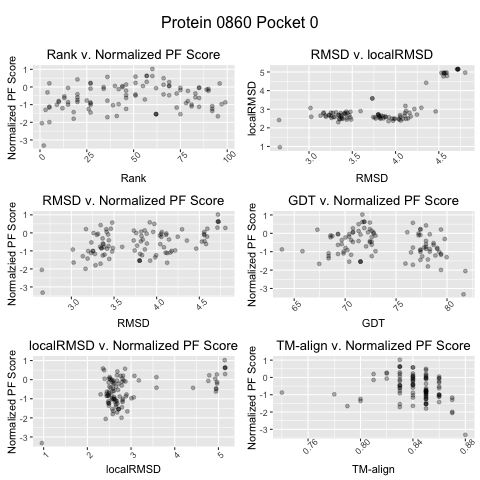

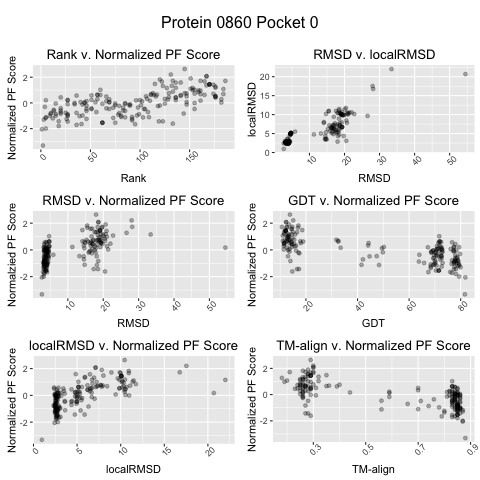


| Server ID | Server name | Model | PF-Zscore | GDT | CASP rank |
| --- | --- | --- | --- | --- | --- |
| 005 | BAKER-ROSETTASERVER | 5 | -3.318 | 81.62 | 2 |
| 005 | BAKER-ROSETTASERVER | 1 | -2.052 | 81.8 | 1 |
| 220 | GOAL | 1 | -1.992 | 79.41 | 5 |
| 275 | slbio | 3 | -1.799 | 78.12 | 18 |
| 359 | Atome2_CBS | 1 | -1.711 | 76.65 | 36 |
| 251 | myprotein-me | 2 | -1.658 | 67.46 | 95 |
| 005 | BAKER-ROSETTASERVER | 3 | -1.645 | 75.92 | 41 |
| 432 | Pcons-net | 5 | -1.611 | 14.71 | 133 |
| 287 | MULTICOM-CLUSTER | 2 | -1.541 | 70.4 | 75 |
| 405 | IntFOLD4 | 3 | -1.539 | 71.51 | 62 |
| 405 | IntFOLD4 | 4 | -1.539 | 71.51 | 62 |
| 405 | IntFOLD4 | 5 | -1.539 | 71.51 | 62 |
| 275 | slbio | 2 | -1.446 | 77.39 | 27 |
| 321 | GAPF_LNCC_SERVER | 1 | -1.435 | 16.54 | 123 |
| 432 | Pcons-net | 3 | -1.432 | 17.28 | 119 |
| 220 | GOAL | 5 | -1.427 | 78.12 | 18 |
| 251 | myprotein-me | 4 | -1.358 | 68.38 | 92 |
| 430 | GOAL_COMPLEX | 1 | -1.296 | 79.96 | 3 |
| 275 | slbio | 4 | -1.253 | 77.57 | 26 |
| 345 | MULTICOM-NOVEL | 4 | -1.242 | 72.61 | 46 |
| 251 | myprotein-me | 3 | -1.239 | 68.38 | 92 |
| 313 | HHGG | 4 | -1.204 | 78.86 | 12 |
| 287 | MULTICOM-CLUSTER | 3 | -1.167 | 70.59 | 73 |
| 430 | GOAL_COMPLEX | 3 | -1.166 | 79.41 | 5 |
| 407 | Distill | 3 | -1.126 | 48.35 | 103 |
| 421 | MUfold2 | 2 | -1.123 | 69.3 | 85 |
| 430 | GOAL_COMPLEX | 4 | -1.114 | 79.41 | 5 |
| 357 | FLOUDAS_SERVER | 1 | -1.110 | 41.54 | 109 |
| 258 | MUfold1 | 2 | -1.094 | 69.85 | 81 |
| 048 | ToyPred_email | 1 | -1.086 | 68.38 | 92 |

T0882


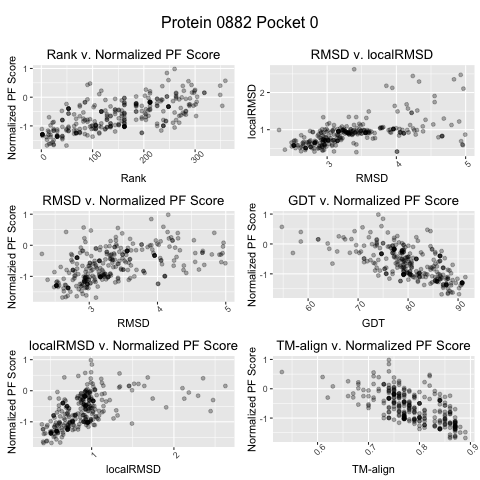

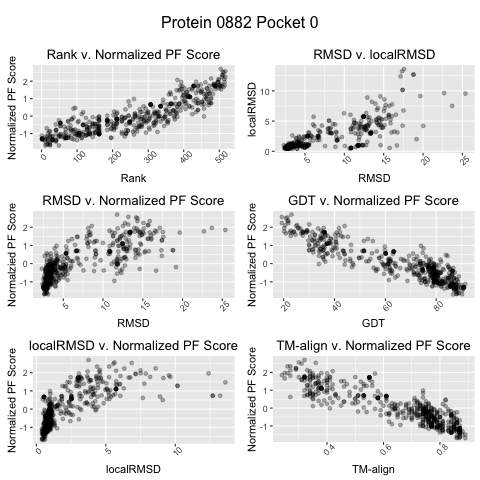


| Server ID | Server name | Model | PF-Zscore | GDT | CASP rank |
| --- | --- | --- | --- | --- | --- |
| 011 | LEE | 3 | -1.684 | 86.71 | 49 |
| 450 | LEEab | 1 | -1.680 | 90.19 | 11 |
| 220 | GOAL | 5 | -1.666 | 87.97 | 24 |
| 011 | LEE | 1 | -1.640 | 87.97 | 24 |
| 011 | LEE | 2 | -1.626 | 88.92 | 15 |
| 011 | LEE | 4 | -1.612 | 88.61 | 18 |
| 450 | LEEab | 2 | -1.565 | 88.29 | 22 |
| 384 | wfMESHI-Seok | 2 | -1.494 | 87.97 | 24 |
| 450 | LEEab | 3 | -1.480 | 90.19 | 11 |
| 384 | wfMESHI-Seok | 3 | -1.464 | 87.03 | 41 |
| 303 | wfMESHI-TIGRESS | 5 | -1.413 | 87.03 | 41 |
| 303 | wfMESHI-TIGRESS | 2 | -1.387 | 90.51 | 10 |
| 450 | LEEab | 4 | -1.382 | 88.61 | 18 |
| 073 | Wallner | 4 | -1.373 | 83.23 | 100 |
| 005 | BAKER-ROSETTASERVER | 1 | -1.371 | 87.66 | 31 |
| 067 | wfRstta-PQ2-Seder | 3 | -1.371 | 87.66 | 31 |
| 114 | Kloczkowski | 4 | -1.371 | 87.66 | 31 |
| 203 | ProQ2 | 2 | -1.371 | 87.66 | 31 |
| 417 | VoroMQA-select | 2 | -1.371 | 87.66 | 31 |
| 102 | Kiharalab | 4 | -1.357 | 87.03 | 41 |
| 220 | GOAL | 3 | -1.357 | 87.03 | 41 |
| 384 | wfMESHI-Seok | 5 | -1.347 | 90.82 | 2 |
| 498 | AP_1 | 3 | -1.338 | 87.66 | 31 |
| 060 | KIAS-Gdansk | 5 | -1.328 | 87.66 | 31 |
| 073 | Wallner | 3 | -1.328 | 84.49 | 90 |
| 456 | wfRosetta-Wallner | 1 | -1.313 | 88.61 | 18 |
| 384 | wfMESHI-Seok | 1 | -1.303 | 89.56 | 14 |
| 005 | BAKER-ROSETTASERVER | 2 | -1.298 | 90.82 | 2 |
| 067 | wfRstta-PQ2-Seder | 1 | -1.298 | 90.82 | 2 |
| 114 | Kloczkowski | 3 | -1.298 | 90.82 | 2 |

T0920-1


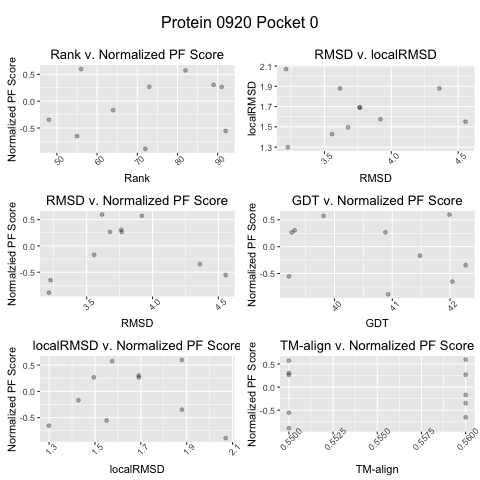

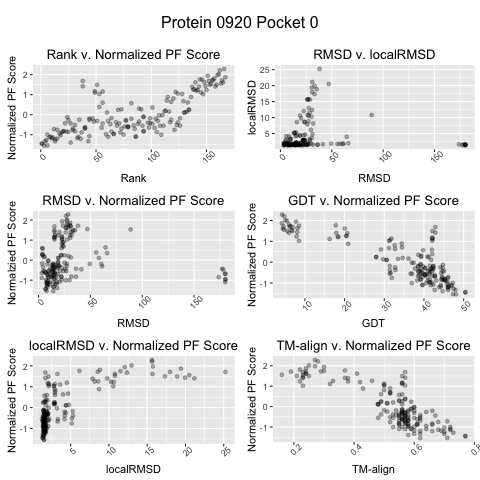


| Server ID | Server name | Model | PF-Zscore | GDT | CASP rank |
| --- | --- | --- | --- | --- | --- |
| 220 | GOAL | 3 | -1.542 | 48.06 | 3 |
| 077 | FALCON_TOPO | 4 | -1.535 | 47.27 | 6 |
| 048 | ToyPred_email | 1 | -1.445 | 50.42 | 1 |
| 092 | RaptorX | 1 | -1.445 | 50.42 | 1 |
| 345 | MULTICOM-NOVEL | 1 | -1.392 | 44.68 | 27 |
| 425 | FALCON_TOPOX | 3 | -1.335 | 46.06 | 13 |
| 479 | Zhang-Server | 1 | -1.329 | 42.69 | 43 |
| 220 | GOAL | 4 | -1.328 | 47.92 | 4 |
| 077 | FALCON_TOPO | 3 | -1.272 | 47.45 | 5 |
| 077 | FALCON_TOPO | 1 | -1.228 | 46.06 | 13 |
| 016 | FFAS-3D | 1 | -1.202 | 43.84 | 36 |
| 236 | MULTICOM-CONSTRUCT | 5 | -1.194 | 45.97 | 15 |
| 077 | FALCON_TOPO | 2 | -1.182 | 46.25 | 10 |
| 005 | BAKER-ROSETTASERVER | 4 | -1.170 | 44.26 | 33 |
| 425 | FALCON_TOPOX | 4 | -1.140 | 46.16 | 11 |
| 250 | Seok-server | 1 | -1.128 | 46.99 | 8 |
| 183 | QUARK | 1 | -1.100 | 41.71 | 61 |
| 005 | BAKER-ROSETTASERVER | 3 | -1.099 | 44.21 | 34 |
| 405 | IntFOLD4 | 3 | -1.095 | 39.63 | 86 |
| 425 | FALCON_TOPOX | 5 | -1.092 | 46.85 | 9 |
| 382 | RBO_Aleph | 4 | -1.087 | 39.63 | 86 |
| 005 | BAKER-ROSETTASERVER | 1 | -1.071 | 42.36 | 44 |
| 425 | FALCON_TOPOX | 1 | -1.030 | 45.83 | 18 |
| 183 | QUARK | 4 | -1.004 | 36.94 | 109 |
| 077 | FALCON_TOPO | 5 | -0.995 | 47.27 | 6 |
| 407 | Distill | 4 | -0.994 | 40.6 | 75 |
| 407 | Distill | 2 | -0.978 | 41.94 | 59 |
| 405 | IntFOLD4 | 4 | -0.969 | 41.44 | 66 |
| 405 | IntFOLD4 | 5 | -0.958 | 41.44 | 66 |
| 287 | MULTICOM-CLUSTER | 2 | -0.940 | 45.93 | 17 |

T0920-1


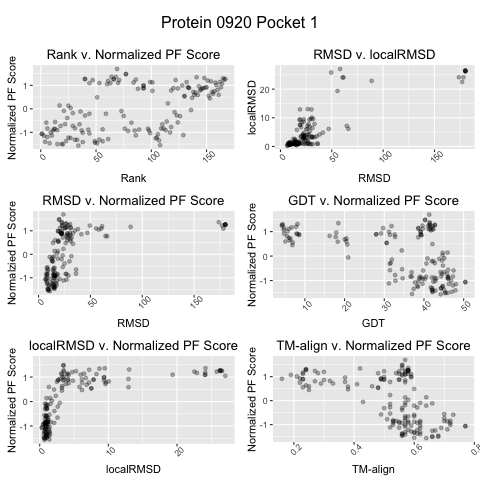


| Server ID | Server name | Model | PF-Zscore | GDT | CASP rank |
| --- | --- | --- | --- | --- | --- |
| 005 | BAKER-ROSETTASERVER | 3 | -1.562 | 44.21 | 34 |
| 220 | GOAL | 1 | -1.548 | 36.99 | 108 |
| 250 | Seok-server | 3 | -1.505 | 45.32 | 20 |
| 250 | Seok-server | 5 | -1.500 | 45.97 | 15 |
| 250 | Seok-server | 2 | -1.498 | 45.42 | 19 |
| 313 | HHGG | 5 | -1.468 | 42.13 | 51 |
| 250 | Seok-server | 1 | -1.463 | 46.99 | 8 |
| 313 | HHGG | 2 | -1.439 | 42.18 | 49 |
| 349 | HHPred1 | 1 | -1.414 | 42.87 | 38 |
| 220 | GOAL | 4 | -1.403 | 47.92 | 4 |
| 313 | HHGG | 3 | -1.380 | 41.99 | 56 |
| 313 | HHGG | 4 | -1.376 | 42.18 | 49 |
| 236 | MULTICOM-CONSTRUCT | 2 | -1.373 | 45.05 | 23 |
| 220 | GOAL | 2 | -1.341 | 37.41 | 107 |
| 005 | BAKER-ROSETTASERVER | 4 | -1.317 | 44.26 | 33 |
| 250 | Seok-server | 4 | -1.316 | 44.35 | 31 |
| 313 | HHGG | 1 | -1.311 | 42.08 | 53 |
| 183 | QUARK | 1 | -1.285 | 41.71 | 61 |
| 220 | GOAL | 5 | -1.254 | 38.47 | 104 |
| 236 | MULTICOM-CONSTRUCT | 3 | -1.244 | 44.68 | 27 |
| 287 | MULTICOM-CLUSTER | 5 | -1.223 | 44.72 | 25 |
| 444 | BhageerathH-Plus | 5 | -1.222 | 38.33 | 105 |
| 077 | FALCON_TOPO | 4 | -1.197 | 47.27 | 6 |
| 220 | GOAL | 3 | -1.147 | 48.06 | 3 |
| 077 | FALCON_TOPO | 2 | -1.136 | 46.25 | 10 |
| 382 | RBO_Aleph | 4 | -1.127 | 39.63 | 86 |
| 275 | slbio | 5 | -1.115 | 31.16 | 129 |
| 275 | slbio | 4 | -1.077 | 37.73 | 106 |
| 048 | ToyPred_email | 1 | -1.063 | 50.42 | 1 |
| 092 | RaptorX | 1 | -1.063 | 50.42 | 1 |

Table S6: Servers that predict best functional relevant models (key patches).

| T0860 | | T0882 | | T0920-1 | | T0920-2 | |
| --- | --- | --- | --- | --- | --- | --- | --- |
| Server /model | | Server /model | | Server /model | | Server /model | |
| BAKER-ROSETTASERVER | 5 | LEE | 3 | GOAL | 3 | BAKER-ROSETTASERVER | 3 |
| BAKER-ROSETTASERVER | 1 | LEEab | 1 | FALCON_TOPO | 4 | GOAL | 1 |
| GOAL | 1 | GOAL | 5 | ToyPred_email | 1 | Seok-server | 3 |
| slbio | 3 | LEE | 1 | RaptorX | 1 | Seok-server | 5 |
| Atome2_CBS | 1 | LEE | 2 | MULTICOM-NOVEL | 1 | Seok-server | 2 |
| myprotein-me | 2 | LEE | 4 | FALCON_TOPOX | 3 | HHGG | 5 |
| BAKER-ROSETTASERVER | 3 | LEEab | 2 | Zhang-Server | 1 | Seok-server | 1 |
| Pcons-net | 5 | wfMESHI-Seok | 2 | GOAL | 4 | HHGG | 2 |
| MULTICOM-CLUSTER | 2 | LEEab | 3 | FALCON_TOPO | 3 | HHPred1 | 1 |
| IntFOLD4 | 3 | wfMESHI-Seok | 3 | FALCON_TOPO | 1 | GOAL | 4 |
| IntFOLD4 | 4 | wfMESHI-TIGRESS | 5 | FFAS-3D | 1 | HHGG | 3 |
| IntFOLD4 | 5 | wfMESHI-TIGRESS | 2 | MULTICOM-CONSTRUCT | 5 | HHGG | 4 |
| slbio | 2 | LEEab | 4 | FALCON_TOPO | 2 | MULTICOM-CONSTRUCT | 2 |
| GAPF_LNCC_SERVER | 1 | Wallner | 4 | BAKER-ROSETTASERVER | 4 | GOAL | 2 |
| Pcons-net | 3 | BAKER-ROSETTASERVER | 1 | FALCON_TOPOX | 4 | BAKER-ROSETTASERVER | 4 |
| GOAL | 5 | wfRstta-PQ2-Seder | 3 | Seok-server | 1 | Seok-server | 4 |
| myprotein-me | 4 | Kloczkowski | 4 | QUARK | 1 | HHGG | 1 |
| GOAL_COMPLEX | 1 | ProQ2 | 2 | BAKER-ROSETTASERVER | 3 | QUARK | 1 |
| slbio | 4 | VoroMQA-select | 2 | IntFOLD4 | 3 | GOAL | 5 |
| MULTICOM-NOVEL | 4 | Kiharalab | 4 | FALCON_TOPOX | 5 | MULTICOM-CONSTRUCT | 3 |
| myprotein-me | 3 | GOAL | 3 | RBO_Aleph | 4 | MULTICOM-CLUSTER | 5 |
| HHGG | 4 | wfMESHI-Seok | 5 | BAKER-ROSETTASERVER | 1 | BhageerathH-Plus | 5 |
| MULTICOM-CLUSTER | 3 | AP_1 | 3 | FALCON_TOPOX | 1 | FALCON_TOPO | 4 |
| GOAL_COMPLEX | 3 | KIAS-Gdansk | 5 | QUARK | 4 | GOAL | 3 |
| Distill | 3 | Wallner | 3 | FALCON_TOPO | 5 | FALCON_TOPO | 2 |
| MUfold2 | 2 | wfRosetta-Wallner | 1 | Distill | 4 | RBO_Aleph | 4 |
| GOAL_COMPLEX | 4 | wfMESHI-Seok | 1 | Distill | 2 | slbio | 5 |
| FLOUDAS_SERVER | 1 | BAKER-ROSETTASERVER | 2 | IntFOLD4 | 4 | slbio | 4 |
| MUfold1 | 2 | wfRstta-PQ2-Seder | 1 | IntFOLD4 | 5 | ToyPred_email | 1 |
| ToyPred_email | 1 | Kloczkowski | 3 | MULTICOM-CLUSTER | 2 | RaptorX | 1 |

Section 2 summary

Table S7. Servers that frequently picked in top 30 predictions (holo sites)

| Server_ID | Server Name | Count / Total |
| --- | --- | --- |
| 236 | MULTICOM-CONSTRUCT | 5\6 |
| 405 | IntFOLD4 | 5\6 |
| 349 | HHPred1 | 4\6 |
| 313 | HHGG | 3\6 |
| 166 | FFAS03 | 3\6 |
| 220 | GOAL | 3\6 |
| 446 | YASARA | 3\6 |
| 005 | BAKER-ROSETTASERVER | 3\6 |
| 077 | FALCON_TOPO | 3\6 |
| 287 | MULTICOM-CLUSTER | 3\6 |
| 016 | FFAS-3D | 3\6 |
| 275 | slbio | 3\6 |
| 048 | ToyPred_email | 2\6 |
| 479 | Zhang-Server | 2\6 |
| 345 | MULTICOM-NOVEL | 2\6 |
| 382 | RBO_Aleph | 2\6 |
| 250 | Seok-server | 2\6 |
| 425 | FALCON_TOPOX | 2\6 |
| 119 | HHPred0 | 2\6 |

Table S7. Servers that frequently picked in top 30 predictions (apo sites)

| Server_ID | Server Name | Count / Total |
| --- | --- | --- |
| 005 | BAKER-ROSETTASERVER | 4\6 |
| 067 | wfRstta-PQ2-Seder | 4\6 |
| 456 | wfRosetta-Wallner | 3\6 |
| 119 | HHPred0 | 2\6 |
| 349 | HHPred1 | 2\6 |
| 064 | Jones-UCL | 2\6 |
| 393 | MESHI | 2\6 |
| 446 | YASARA | 2\6 |
| 252 | wfRosetta-ProQ-ModF6 | 2\6 |
| 498 | AP_1 | 2\6 |
| 077 | FALCON_TOPO | 2\6 |
| 439 | MULTICOM | 2\6 |
| 220 | GOAL | 2\6 |
| 417 | VoroMQA-select | 2\6 |
| 247 | BAKER | 2\6 |
| 232 | Chicken_George | 2\6 |
| 114 | Kloczkowski | 2\6 |
| 102 | Kiharalab | 2\6 |
| 243 | Seok-refine | 2\6 |
| 464 | tsspred2 | 2\6 |
| 073 | Wallner | 2\6 |
| 411 | Pcomb-domain | 2\6 |
| 303 | wfMESHI-TIGRESS | 2\6 |

Table S8. Servers that frequently picked in top 30 predictions (key patches)

| Server_ID | Server Name | Count / Total |
| --- | --- | --- |
| 048 | ToyPred_email | 3\4 |
| 220 | GOAL | 3\4 |
| 005 | BAKER-ROSETTASERVER | 3\4 |
| 382 | RBO_Aleph | 2\4 |
| 183 | QUARK | 2\4 |
| 250 | Seok-server | 2\4 |
| 092 | RaptorX | 2\4 |
| 077 | FALCON_TOPO | 2\4 |
| 313 | HHGG | 2\4 |
| 405 | IntFOLD4 | 2\4 |
| 287 | MULTICOM-CLUSTER | 2\4 |
| 275 | slbio | 2\4 |

**Section 3: Collaboration with protein assembly assessment.**

Table S9. We compared the PocketFEATURE scores on T0893 with protein assembly scores (from Capitani research group). There are only three models that overlap between our assessment. Model 430-1 is ranked best in protein assembly prediction.

|  |  | Protein assembly scores (Capitani group) | | | CASP | Functional site (Altman group) |
| --- | --- | --- | --- | --- | --- | --- |
| Model | Server | F1 | Prec. | Recall | GDT | PF-score |
| 430-1 | GOAL_COMPLEX | 46.5 | 37.6 | 60.8 | 49.8 | -15.4 |
| 495-1 | Seok-assembly | 10.2 | 11.2 | 9.3 | 53.6 | -10.7 |
| 005-1 | BAKER-ROSETTASERVER | 8.4 | 6.6 | 11.3 | 61.5 | -11.1 |

Figure S2. Interestingly, 430-1 is also ranked in top 30 predictions when we assess its ADP binding site. The ADP binding site of target T0893 involves interaction of the catalytic domain (CA), green to red below, and the helical dimerization (DHp) domain, blue to cyan below. This figure suggests that PF is useful in this particular case to identify correct domain orientation and interaction and can be an interesting score to assess protein-protein interactions with active pockets.


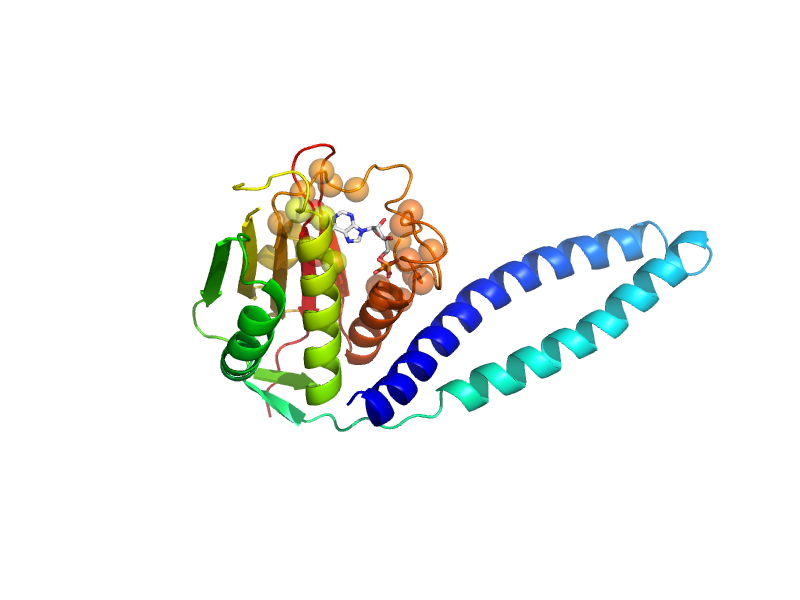


Figure S3. The median GDT_TS for T0945 predictions is 41.22 (rank 41 out of 71 regular targets), which is close to the average of 45.85. This indicates that T0945 is not a particularly hard or easy target to predict.

Distribution of predicted relative solvent accessibility values for disease causing variants in T0945 (DPAGT1) and the correct value (red dot). Average standard deviation: 0.14, average root mean-squared-error: 0.20. There is no significant difference between these values and the metrics for residues affected by VUS (0.15 and 0.24)


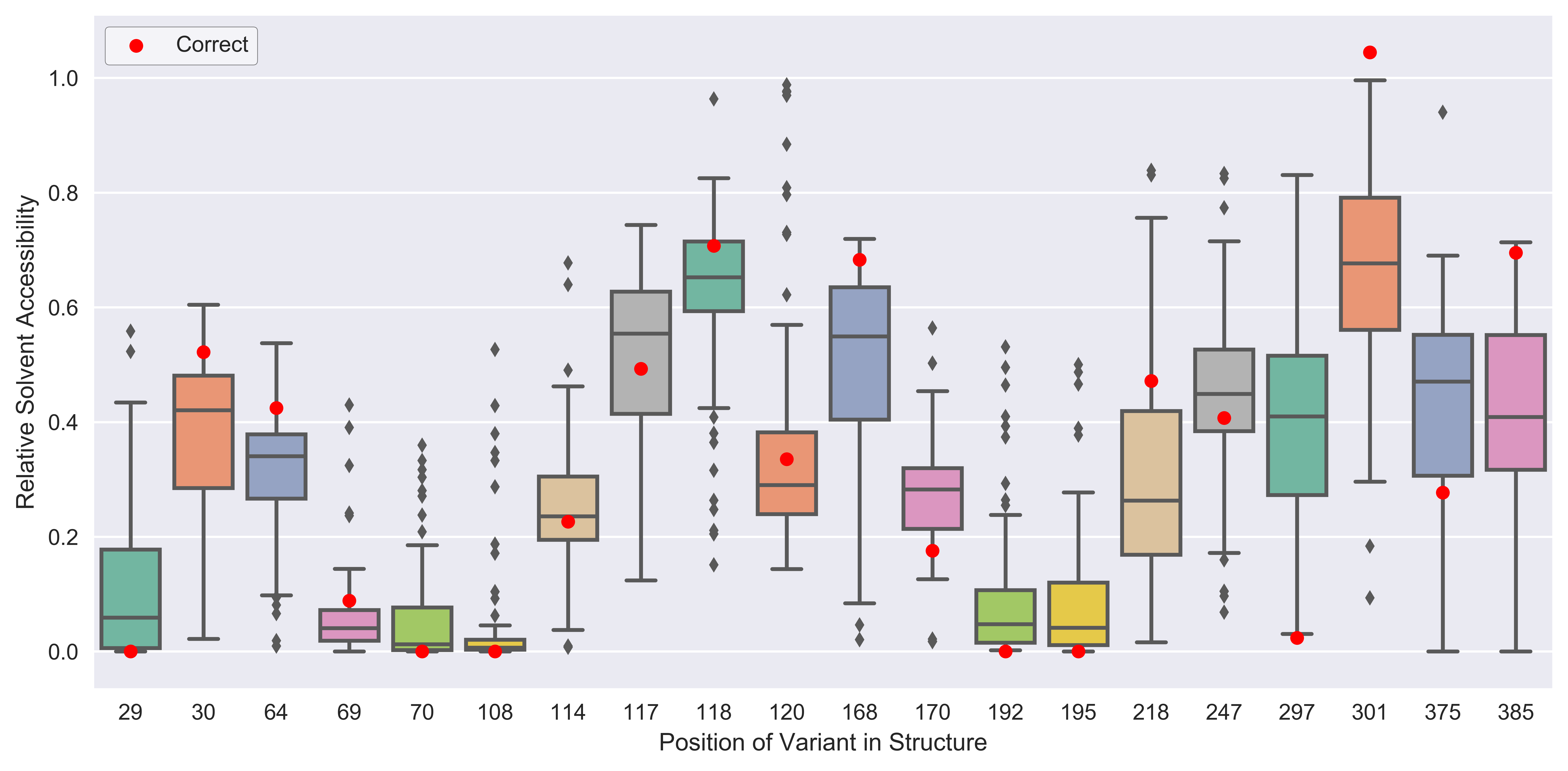


Table S10. SIFT Scores for variants in T0945. Fifteen out of twenty variants (75%) annotated as being pathogenic are predicted to be damaging (SIFT Score <= 0.05). 36 out of 75 (48%) substitutions are predicted to be tolerated (SIFT Score > 0.05) and labeled as variant of unknown significance (VUS).

| Position of Variant in Structure | SIFT Score | Clinical Significance |  | Position of Variant in Structure | SIFT Score | Clinical Significance |
| --- | --- | --- | --- | --- | --- | --- |
| 30 | 0.04 | Pathogenic |  | 151 | 0 | VUS |
| 31 | 0.04 | Pathogenic |  | 162 | 0.31 | VUS |
| 65 | 0.23 | Pathogenic |  | 163 | 0 | VUS |
| 70 | 0.01 | Pathogenic |  | 170 | 0 | VUS |
| 71 | 0.02 | Pathogenic |  | 179 | 0 | VUS |
| 109 | 0.46 | Pathogenic |  | 192 | 0 | VUS |
| 115 | 0.41 | Pathogenic |  | 193 | 0 | VUS |
| 118 | 0.12 | Pathogenic |  | 205 | 0.77 | VUS |
| 119 | 0 | Pathogenic |  | 211 | 0.66 | VUS |
| 121 | 0.01 | Pathogenic |  | 224 | 0 | VUS |
| 169 | 0 | Pathogenic |  | 235 | 0.08 | VUS |
| 171 | 0 | Pathogenic |  | 237 | 1 | VUS |
| 193 | 0 | Pathogenic |  | 239 | 0.28 | VUS |
| 196 | 0.01 | Pathogenic |  | 242 | 0 | VUS |
| 219 | 0.2 | Pathogenic |  | 244 | 0.01 | VUS |
| 248 | 0 | Pathogenic |  | 247 | 0 | VUS |
| 298 | 0 | Pathogenic |  | 258 | 0 | VUS |
| 302 | 0 | Pathogenic |  | 265 | 0.04 | VUS |
| 376 | 0.01 | Pathogenic |  | 266 | 0.15 | VUS |
| 386 | 0.35 | Pathogenic |  | 274 | 0 | VUS |
| 8 | 0.1 | VUS |  | 278 | 0 | VUS |
| 11 | 0 | VUS |  | 281 | 0 | VUS |
| 14 | 0.94 | VUS |  | 284 | 0.33 | VUS |
| 22 | 0.04 | VUS |  | 290 | 1 | VUS |
| 26 | 0.3 | VUS |  | 296 | 0.04 | VUS |
| 27 | 0.61 | VUS |  | 297 | 0 | VUS |
| 29 | 0.09 | VUS |  | 298 | 0 | VUS |
| 41 | 0.01 | VUS |  | 301 | 0 | VUS |
| 42 | 1 | VUS |  | 303 | 1 | VUS |
| 48 | 0 | VUS |  | 309 | 0.11 | VUS |
| 50 | 0.58 | VUS |  | 316 | 1 | VUS |
| 52 | 0.09 | VUS |  | 324 | 0.22 | VUS |
| 60 | 0 | VUS |  | 326 | 0.36 | VUS |
| 61 | 0 | VUS |  | 330 | 0.21 | VUS |
| 62 | 0.29 | VUS |  | 332 | 0.37 | VUS |
| 70 | 0.01 | VUS |  | 340 | 0.22 | VUS |
| 75 | 0 | VUS |  | 342 | 0.06 | VUS |
| 105 | 0.1 | VUS |  | 348 | 0.64 | VUS |
| 111 | 0 | VUS |  | 350 | 0.05 | VUS |
| 114 | 0.06 | VUS |  | 371 | 1 | VUS |
| 115 | 0.02 | VUS |  | 375 | 0.01 | VUS |
| 117 | 0.91 | VUS |  | 377 | 0.61 | VUS |
| 120 | 0 | VUS |  | 380 | 0.05 | VUS |
| 121 | 0 | VUS |  | 393 | 0.61 | VUS |
| 131 | 0.03 | VUS |  | 395 | 0.17 | VUS |
| 140 | 0 | VUS |  | 398 | 0 | VUS |
| 141 | 0.01 | VUS |  | 401 | 0.02 | VUS |
| 143 | 0.17 | VUS |  |  |  |  |

Table S11. We compared PF-scores of the 20 mutation patches with GDT scores and AccError values (From Mooney group). In general, the AccError does not correlate with GDT, nor PF-scores.

| Target_ID | Variants | AA | PF-scores range | correlation  PF - GDT | Correlation AccError- GDT |
| --- | --- | --- | --- | --- | --- |
| T0945 | 109 | M | (-12.567, -2.601) | -0.9021 | -0.5785 |
| T0945 | 121 | L | (-6.952, -1.419) | -0.8134 | -0.3333 |
| T0945 | 30 | I | (-9.629, -2.23) | -0.8310 | -0.2664 |
| T0945 | 169 | L | (-5.92, -1.128) | -0.7974 | -0.8036 |
| T0945 | 70 | I | (-9.891, -1.911) | -0.7649 | -0.3646 |
| T0945 | 118 | V | (-6.642, -0.889) | -0.8350 | -0.4421 |
| T0945 | 171 | Y | (-9.153, -1.894) | -0.8245 | -0.4240 |
| T0945 | 196 | A | (-11.089, -1.822) | -0.8800 | -0.7044 |
| T0945 | 115 | A | (-8.454, -1.262) | -0.9053 | -0.4870 |
| T0945 | 65 | A | (-8.045, -1.254) | -0.8017 | -0.5817 |
| T0945 | 119 | L | (-4.111, -0.481) | -0.7671 | -0.7434 |
| T0945 | 71 | L | (-7.232, -1.391) | -0.6840 | -0.5357 |
| T0945 | 248 | R | (-9.05, -1.836) | -0.8193 | -0.6124 |
| T0945 | 376 | H | (-5.006, -0.931) | -0.5519 | 0.0752 |
| T0945 | 386 | L | (-5.343, -0.271) | -0.6516 | -0.4750 |
| T0945 | 193 | G | (-9.098, -1.12) | -0.7644 | -0.4611 |
| T0945 | 302 | R | (-1.737, 100) | -0.1246 | -0.2841 |
| T0945 | 298 | I | (-4.066, -0.791) | -0.5887 | 0.2211 |
| T0945 | 31 | P | (-8.461, -2.167) | -0.7960 | -0.3264 |
| T0945 | 219 | R | (-3.84, -0.583) | -0.2780 | 0.1681 |

**Section 4: Method description**

PocketFEATURE contains two essential modules to evaluate and compare physicochemical properties of a single or a cluster of functional centers. The two modules are:

Similarity measure between two FEATURE microenvironments

Given a pair of FEATURE microenvironments (A and B) derived from two different sites, we calculate an adjusted Tanimoto coefficient based on the presence/absence of similar properties. We compute a single standard deviation (STD) for each of the 480 properties across a random set of FEATURE microenvironments. Two microenvironments have a “similar property” if they differ by less than one STD for the given property. Given A and B, c is the number of “similar properties”; a and b are the numbers of non-zero properties in A and B, respectively; the denominator is the total number of unique properties that are non-zero in A or B or both (a+b-c); then the Tanimoto similarity is as follows:

We make observations of the background distributions of Tc scores between microenvironments from different sites. We compile a dataset of 1160 sites from a non-redundant set of 3D structures in PDB using these filters: (1) structures were solved using X-ray diffraction at resolutions higher than 2.0 Angstrom; (2) no two structures have greater than 40% sequence identity; (3) specifically bound small molecule ligands have more than the heavy atoms. The binding residues are defined as those having any atom within 6 Angstroms of the ligand molecules, resulting in a total of 22008 microenvironments. There are 242 possible types of pairs between 22 types of microenvironments centered on 20 residues types (two centers for residue W and Y), but not all of these are likely to be matched. For computational efficiency, we group residues by physical properties in order to avoid comparisons that are unlikely to yield high similarity scores. The comparisons are limited to pairs of microenvironments that fall within the same groups: positively charged (R H K), negatively charged (D E), polar (S T Q N W1 Y1), non-polar (A C G I L M P V) and aromatic (W2 Y2 F). This produces 72 microenvironment-pairs (out of the 200 possible) that we check for high similarity scores. Given each microenvironment-pair, we derive Tc scores from the above dataset and fit these score into normal distribution.

Comparing two binding sites

Given the FEATURE microenvironments from two binding sites, we exhaustively calculate the raw Tc scores of all permissible microenvironment-pairs. We then normalize the Tc scores using the background frequency:

Tc0 is the Tc score at the mode on the fitted cumulative distribution function (CDF) of a given type of microenvironment-pair (See Background calculation). The normalized value, S(Tc), measures the similarity between two microenvironments and is thus the microenvironment similarity score.

We search for the mutual best-scoring microenvironment-pairs between two binding sites and assign alignment to such pairs using an cutoff of S(Tc) less than -0.3. For example, between site A (microenvironments A1, A2, A3, A4 and A5) and site B (microenvironments B1, B2, B3, B4 and B5), we align A1 to B1 only when (1) S(Tc) between A1 and B1 is smaller than those between A1 and B1, B2, B4 B5, also smaller than those between B1 and A2, A3, A4, A5; (2) S(Tc) between A1 and B1 is smaller than -0.3. The sum of all aligned microenvironment-pairs is the overall similarity score between two binding sites, and is termed the binding site similarity score. We can vary the cutoff for S(Tc) to change the precision and resolution of the comparison.
